# Identification and Experimental Verification Reveal SLC2A3 Associated With Prognosis and Immune Infiltration in Colon Adenocarcinoma

**DOI:** 10.1155/mi/8383379

**Published:** 2026-04-24

**Authors:** Zhenyuan Zhao, Kun Ju, Jiajun Lv, Zidong Lu, Lei Luo

**Affiliations:** ^1^ Department of Vascular Surgery, The Affiliated Hospital of Qingdao University, 266071, Qingdao, China, qdu.edu.cn; ^2^ Department of Emergency, The Affiliated Hospital of Qingdao University, 266071, Qingdao, China, qdu.edu.cn; ^3^ Department of Gastrointestinal Surgery, The Affiliated Hospital of Qingdao University, 266071, Qingdao, China, qdu.edu.cn

**Keywords:** colon adenocarcinoma, immune infiltration, molecular subtype, parthanatos, SLC2A3

## Abstract

Colon adenocarcinoma (COAD) exhibits marked molecular heterogeneity that limits accurate prognostic prediction and therapeutic stratification. Parthanatos, a poly (ADP‐ribose) polymerase‐1 (PARP1)‐dependent form of regulated cell death, has been implicated in tumor biology, yet its relevance in COAD remains poorly understood. In this study, we systematically characterized parthanatos‐associated genes (PAGs) using integrated transcriptomic analyses from The Cancer Genome Atlas (TCGA) and Gene Expression Omnibus (GEO) cohorts. Distinct parthanatos‐related molecular subtypes with significantly different clinical outcomes and immune landscapes were identified. A parthanatos‐based prognostic scoring system was constructed using machine learning algorithms and validated in an independent cohort, demonstrating robust predictive performance and independent prognostic value. High PAG scores were associated with advanced disease stage, altered immune infiltration, increased tumor mutation burden (TMB), and differential sensitivity to chemotherapy and predicted immunotherapy response. Single‐cell RNA sequencing further revealed broad cellular expression of PAG, while preliminary in vitro experiments suggested that solute carrier family 2 member 3 (SLC2A3), the most prominent risk gene in the prognostic model, may promote COAD cell proliferation and invasion, although these findings require validation in additional models. Collectively, our findings establish parthanatos as a clinically relevant regulatory axis in COAD and provide a novel framework for prognosis assessment and therapeutic targeting.

## 1. Introduction

Colon adenocarcinoma (COAD) remains one of the most prevalent and lethal malignancies worldwide, posing a substantial public health burden despite advances in screening and therapeutic strategies [1]. Although current clinical management, including surgery, chemotherapy, radiation, and targeted or immunotherapeutic approaches, has improved outcomes, patient prognoses still vary markedly due to the pronounced molecular and cellular heterogeneity of COAD [2–4]. Traditional staging systems, such as TNM classification, often fail to fully capture this complexity, leading to suboptimal prediction of treatment response and survival [5]. Therefore, there is a critical need for more refined risk‐stratification tools that integrate molecular characteristics to better delineate patient subgroups, guide individualized therapy, and enhance clinical decision‐making.

Parthanatos is a distinct form of regulated cell death that is mechanistically and morphologically different from apoptosis, necroptosis, and other known cell death modalities [6]. It is primarily triggered by excessive activation of poly (ADP‐ribose) polymerase‐1 (PARP1) in response to severe DNA damage or oxidative stress [7, 8]. Hyperactivation of PARP1 leads to the accumulation of poly (ADP‐ribose) (PAR) polymers, which subsequently induce mitochondrial dysfunction and promote the translocation of apoptosis‐inducing factor (AIF) from the mitochondria to the nucleus. Nuclear AIF causes large‐scale DNA fragmentation and chromatin condensation in a caspase‐independent manner, ultimately resulting in irreversible cell death [9, 10]. Given its unique molecular features and signaling cascade, parthanatos is increasingly recognized as a critical component of the regulated cell death network [11].

Accumulating evidence suggests that parthanatos plays a complex and context‐dependent role in tumor biology [12, 13]. On one hand, activation of parthanatos may function as a tumor‐suppressive mechanism by eliminating malignant cells exposed to overwhelming genotoxic stress, thereby enhancing the cytotoxic effects of chemotherapy and radiotherapy [14]. On the other hand, dysregulation of parthanatos‐related pathways, including aberrant PARP1 activity, impaired AIF translocation, or altered DNA damage responses, may enable cancer cells to evade cell death and acquire therapeutic resistance [6, 14]. Moreover, emerging studies indicate that parthanatos may influence the tumor immune microenvironment and inflammatory signaling through PARP‐dependent metabolic and transcriptional reprogramming [14]. These findings highlight parthanatos as a double‐edged sword in cancer progression and treatment, underscoring its potential as both a prognostic indicator and a therapeutic target.

In this study, we systematically investigated the role of parthanatos in COAD through an integrative multiomics framework. By comprehensively characterizing parthanatos‐associated genes (PAGs), we identified distinct molecular subtypes with heterogeneous clinical outcomes, metabolic features, and immune landscapes. We further constructed and validated a parthanatos‐based scoring model to stratify patients according to prognosis and therapeutic responsiveness. In addition, we explored the biological relevance of key PAG, with particular emphasis on solute carrier family 2 member 3 (SLC2A3), through in vitro functional assays. Collectively, this study provides a global view of parthanatos‐related molecular heterogeneity in COAD and establishes a foundation for understanding its potential clinical and biological implications.

## 2. Materials and Methods

### 2.1. Data Collection

In this study, transcriptomic expression data and clinical baseline information for all samples were obtained from publicly available databases, including the Gene Expression Omnibus (GEO, https://www.ncbi.nlm.nih.gov/geo/) and The Cancer Genome Atlas (TCGA, https://portal.gdc.cancer.gov/). For the TCGA dataset, transcriptomic expression matrices of normal tissues and COAD samples were annotated using the Perl programming environment. Gene annotation was performed based on the GENCODE database (GRCh38, version 22), and Ensembl gene IDs were mapped to official gene symbols. From the GEO database, two datasets containing both COAD transcriptomic data and complete clinical baseline information (GSE39582 and GSE17538) were selected and downloaded. Both datasets were generated using the GPL570 platform ([HG‐U133_Plus_2] Affymetrix Human Genome U133 Plus 2.0 Array). Probe IDs were annotated and converted to corresponding gene symbols according to platform‐specific annotation files using Perl. In the R programming environment, the “limma” R package was employed to convert the raw count matrix of the TCGA dataset into transcripts per million (TPM) formats. Batch effects and systematic biases arising from different data sources and sequencing platforms were corrected using the “sva” R package, and the TCGA‐COAD and GSE39582‐COAD datasets were merged to construct the training cohort. Samples lacking complete survival information or with overall survival less than 30 days were excluded. Ultimately, 42 normal tissue samples and 417 COAD samples from the TCGA‐COAD dataset, 556 COAD samples from the GSE39582‐COAD dataset, and 200 COAD samples from the GSE17538‐COAD dataset were included for subsequent bioinformatic analyses.

### 2.2. Landscape of Parthanatos‐Related Genes in COAD

PAGs were retrieved from the GeneCards database (https://www.genecards.org/) using the keyword “Parthanatos.” In this study, genes were selected based on their relevance scores and documented associations with parthanatos‐related processes, including PARP1 activation, DNA damage response, and AIF‐mediated cell death. A total of 37 PAG were retained as candidate genes for subsequent analyses (Supporting Information 1: Table S1) [15, 16]. Differential expression analysis of PAG between normal and COAD samples was performed using the “limma” R package. Genes with |fold change| ≥ 1.5 and adjusted *p*‐value < 0.05 were considered differentially expressed. A volcano plot was generated to visualize the expression distribution of PAG across groups. Hierarchical clustering and heatmap visualization of differentially expressed PAG (DE‐PAG) were conducted using the “pheatmap” R package. To investigate potential interactions among DE‐PAG, a protein–protein interaction (PPI) network was constructed using the STRING database (https://string-db.org/) with an interaction confidence score threshold of ≥0.15. Copy number variation (CNV) data of COAD samples were downloaded from the UCSC Xena database (https://xenabrowser.net/datapages/), and the CNV frequency of DE‐PAG was calculated. Somatic mutation profiles were analyzed based on Mutation Annotation Format (MAF) files using the “maftools” R package, and waterfall plots were generated to illustrate mutation frequency and distribution. The chromosomal locations and colocalization patterns of DE‐PAG were visualized using the “RCircos” R package.

### 2.3. Identification of Parthanatos‐Associated Molecular Patterns

Unsupervised consensus clustering of COAD samples in the training cohort was performed based on DE‐PAG expression profiles using the “ConsensusClusterPlus” R package to identify parthanatos‐related molecular subtypes. The optimal number of clusters (*k*) was evaluated for *k* = 2–9 with the following parameters: maxK = 9, reps = 50, pItem = 0.8, pFeature = 1, clustering algorithm = K‐means, and Euclidean distance. The random seed was set to 123456. The optimal *k* was determined based on the cumulative distribution function (CDF) curves and corresponding delta area plots. The CDF curves showed that clustering stability reached a plateau at *k* = 2, while the relative increase in the area under the CDF curve became marginal for *k* > 2, indicating limited gain in stability with additional clusters. Therefore, *k* = 2 was selected as the optimal number of clusters. To explore functional differences among PAG subtypes, gene set variation analysis (GSVA) was performed using the “GSVA” R package based on the Kyoto Encyclopedia of Genes and Genomes (KEGG) pathway reference gene set (“c2.cp.kegg.v7.2.symbols.gmt”), with parameters set as min.sz = 10 and max.sz = 500. The resulting GSVA scores for each pathway were compared among subtypes using linear models implemented in the “limma” R package. Multiple testing correction was performed using the Benjamini–Hochberg (BH) method to control the false discovery rate (FDR). Pathways with adjusted *p*‐value < 0.05 were considered significantly differentially enriched among subtypes.

### 2.4. Immune Microenvironment Infiltration and Immunotherapy Response Prediction

Tumor immune microenvironment features were characterized using transcriptomic data. Genes with low expression (average expression ≤ 0 across) were excluded to reduce noise. The “estimate” R package was used to compute stromal score, immune score, ESTIMATE score, and tumor purity for each sample, representing the relative abundance of stromal and immune components in tumor tissues. Single‐sample gene set enrichment analysis (ssGSEA) was performed using the “GSVA” R package with 23 immune cell marker gene sets and 13 immune function‐related gene sets, quantifying immune cell infiltration and immune functional status. Differences between molecular subtypes were assessed using the Wilcoxon rank‐sum test. Potential responses to immunotherapy were predicted using the Tumor Immune Dysfunction and Exclusion (TIDE) framework and immunophenoscore (IPS) from The Cancer Immunome Atlas (TCIA). TIDE estimates immune evasion and potential efficacy of immune checkpoint inhibitors, while IPS evaluates predicted responsiveness to PD‐1 and CTLA‐4 blockade. To further validate the robustness of immunotherapy predictions, the IMvigor210 cohort (a urothelial carcinoma immunotherapy dataset) was employed as an external immunotherapy validation dataset. This cohort included 348 urothelial carcinoma patients treated with the anti‐PD‐L1 monoclonal antibody atezolizumab, with complete transcriptomic data and clinical response follow‐up. According to original clinical annotations, patients were classified as responders (complete response [CR] and partial response [PR]) or nonresponders (stable disease [SD] and progressive disease [PD]). Differences in immunotherapy response rates among subgroups were compared to evaluate subgroup‐specific efficacy of PD‐L1 blockade therapy.

### 2.5. Biological Features of Parthanatos‐Associated Phenotypes

Based on the previously identified parthanatos molecular subtypes, differential gene expression analysis among different PAG subtypes was conducted using the “limma” R package. Differentially expressed genes (DEGs) were defined as those with |fold change| ≥ 1.5 and adjusted *p*‐value < 0.05. To investigate the potential biological functions and regulatory mechanisms associated with DEGs, Gene Ontology (GO) functional annotation and KEGG pathway enrichment analyses were performed using the “clusterProfiler” R package, with significance adjusted by the BH method for FDR correction. Unsupervised consensus clustering based on DEG expression profiles was performed using the “ConsensusClusterPlus” R package to identify gene subtypes. Optimal clustering was determined by systematically evaluating model stability across different cluster numbers. Principal component analysis (PCA) was performed using the “ggplot2” R package to visualize the distribution and separation of gene subtypes in reduced‐dimensional space. Kaplan–Meier survival curves were plotted using the “survival” R package, and log‐rank tests were applied to compare overall survival among different gene subtypes to assess the relationship between gene subtypes and patient prognosis.

### 2.6. Development and Validation of a Parthanatos‐Related Prognostic Signature

The GSE17538‐COAD dataset was used as an independent external validation cohort. Using gene expression matrices and corresponding overall survival information from the training cohort, multiple machine learning survival models were constructed under a leave‐one‐out cross‐validation (LOOCV) framework to minimize overfitting. Ten algorithms were employed, including LASSO‐Cox, Ridge‐Cox, Elastic Net Cox regression, stepwise Cox regression, CoxBoost, survival support vector machine (survivalSVM), supervised principal components (SuperPCs), partial least squares Cox regression (plsRcox), and gradient boosting machine (GBM)–based Cox regression. Feature selection was performed exclusively within the training set according to each algorithm’s criteria, retaining genes with nonzero coefficients. Hyperparameters were tuned using 10‐fold cross‐validation within the training set, and final models were refitted on the complete training cohort with the optimal parameter sets. Model performance and stability were assessed using the concordance index (C‐index) in both training and external validation cohorts. Models were compared based on C‐index and generalizability to select the most robust and predictive PAG scoring model. Independent prognostic variables were further analyzed using multivariate Cox proportional hazards regression, and hazard coefficients were visualized with the “ggplot2” R package. Based on the median PAG score, both training and validation cohorts were stratified into high‐ and low‐PAG score groups. Overall survival differences between subgroups were evaluated using Kaplan–Meier curves, log‐rank tests, and Cox proportional hazards analysis. Alluvial plots illustrating relationships among parthanatos molecular subtypes, PAG score subgroups, and clinical outcomes were constructed using the “ggalluvial” R package.

### 2.7. Independent Prognostic Analysis and Nomogram Model Construction

Clinical and pathological features were integrated, and PAG score distribution across clinical subgroups was analyzed using the “limma” R package. Univariate and multivariate Cox regression analyses were performed using the “survival” R package to assess the independent prognostic value of PAG scores and clinical variables in both training and validation cohorts. Time‐dependent receiver operating characteristic (ROC) curves and area under the curve (AUC) values at 1‐, 3‐, and 5‐year intervals were calculated using the “survivalROC” R package to evaluate predictive performance. Nomograms integrating PAG scores with clinical variables were constructed using the “rms” R package for predicting 1‐, 3‐, and 5‐year survival probabilities. Calibration curves were plotted with the “regplot” R package to compare predicted and observed survival outcomes. The C‐index of the nomogram was calculated using bootstrap resampling (1000 iterations), and 95% confidence intervals (CIs) were estimated to assess model robustness. The C‐index of PAG scores and clinical variables was computed using “pec” and “rms” R packages to assess discriminatory power and accuracy in survival prediction.

### 2.8. Tumor Mutation Burden (TMB) and Drug Sensitivity Analysis

Microsatellite instability (MSI) status and somatic mutation data (MAF) for COAD samples were obtained from TCGA. Samples were classified as MSI‐high (MSI‐H), MSI‐low (MSI‐L), or microsatellite stable (MSS) according to TCGA criteria. TMB was calculated as the number of somatic nonsynonymous mutations (including missense, nonsense, frameshift insertions, and frameshift deletions) per megabase (Mb) of coding sequence. The “maftools” R package was used to visualize mutation profiles and generate mutation frequency waterfall plots across subgroups. Drug sensitivity was predicted using the “pRRophetic” R package. Ridge regression models were trained on drug response data from the Genomics of Drug Sensitivity in Cancer (GDSC) database, which relates gene expression profiles to half‐maximal inhibitory concentration (IC50) values. Gene expression matrices were preprocessed by removing duplicate probes (avereps) and filtering low‐expressed genes based on average expression. To minimize batch effects arising from different datasets or sequencing platforms, surrogate variable analysis (SVA) was applied prior to prediction. Predicted IC50 values were estimated for each sample, enabling assessment of potential differential drug responses across molecular subgroups.

### 2.9. Single‐Cell RNA Sequencing Data Preprocessing and Annotation

Single‐cell transcriptomic data for three normal and three tumor colon tissues were obtained from GEO (GSE231559), generated on the 10× Genomics platform. Raw data were processed using the Seurat R package. Genes expressed in ≥3 cells and cells with ≥200 detected genes were retained. Cells with 200–10,000 genes and mitochondrial gene content ≤ 20% were kept for downstream analysis. Expression matrices were log‐normalized, and the top 2000 highly variable genes were identified using the “vst” method. PCA was performed, and significant components were determined using “JackStraw” and “ElbowPlot.” Batch effects across samples were corrected using Harmony. Low‐dimensional visualization was performed using t‐SNE and UMAP. Clustering was conducted on the Harmony‐corrected space using K‐nearest neighbor (KNN) and shared nearest neighbor (SNN) graphs, with resolution = 1.2 selected based on clustree analysis. Marker genes for each cluster were identified using Seurat’s FindAllMarkers function (expressed in ≥25% of cells and |log2 fold change| > 0.25). Cell type annotation was performed using “SingleR” with the Human Primary Cell Atlas and CellMarker databases, followed by manual refinement. PAG signature scores were calculated for each cell using the “AddModuleScore” function in Seurat, comparing PAG‐related genes to control gene sets to derive relative enrichment scores. All results were visualized with t‐SNE and UMAP plots.

### 2.10. Cell Culture

The human normal colorectal epithelial cell line NCM460 (*Homo sapiens*, male, derived from normal colorectal mucosal epithelium; RRID: CVCL_0460) was obtained from INCELL Corporation (San Antonio, TX, USA) in May 2024, and the human colorectal cancer (CRC) cell line SW480 (*Homo sapiens*, male, derived from primary COAD; RRID: CVCL_0546) was obtained from the American Type Culture Collection (ATCC, Manassas, VA, USA). Cells were cultured in high‐glucose Dulbecco’s Modified Eagle Medium (DMEM; Gibco) supplemented with 10% fetal bovine serum (FBS; Gibco) and 1% penicillin–streptomycin under standard conditions (37°C, 5% CO_2_, and humidified atmosphere). Cell line authentication was performed by short tandem repeat (STR) profiling prior to experimentation, and the profiles showed ≥80% match with the reference standards. According to the International Cell Line Authentication Committee (ICLAC) and Cellosaurus databases, neither NCM460 nor SW480 has been reported as a commonly misidentified or cross‐contaminated cell line at the time of use. Mycoplasma contamination was routinely tested using PCR‐based detection assays, and all cell cultures were confirmed to be mycoplasma‐free during the experiments. Cells were passaged using 0.25% trypsin–EDTA at 80%–90% confluence with a split ratio of 1:3–1:5. Only cells in the logarithmic growth phase were used, and passage numbers were limited to fewer than 20 generations.

### 2.11. Western Blot Analysis of SLC2A3 Expression

Log‐phase NCM460 and SW480 cells were washed twice with cold PBS, lysed with RIPA buffer containing protease inhibitors (Beyotime, P0013B) on ice for 30 min, and centrifuged at 12,000× g for 15 min. Supernatants were quantified using a BCA assay (Beyotime, P0010). Equal amounts of protein (20–30 μg) were separated by SDS‐PAGE, transferred to PVDF membranes, blocked in 5% milk for 1 h, and incubated with primary antibodies at 4°C overnight: an anti‐SLC2A3 (Cat No. 20403‐1‐AP, Proteintech, 1:1000) and anti‐GAPDH (Cat No. 10494‐1‐AP, Proteintech, 1:5000). Membranes were washed and incubated with HRP‐conjugated secondary antibodies (Cat No. SA00001‐2, Proteintech, 1:5000) for 1 h at room temperature. Protein bands were visualized using ECL (Thermo Fisher Scientific) and quantified with ImageJ. SLC2A3 expression was normalized to internal controls.

### 2.12. siRNA Transfection

SW480 cells were transfected with specific siRNA targeting human SLC2A3 (siSLC2A3) or negative control siRNA (siNC) using Lipofectamine 3000 (Thermo Fisher Scientific) according to manufacturer instructions. The sequence siSLC2A3 was (sequence: 5’‐AGGGAAAUGCCCCACCCUCTT‐3’ and antisense: 5’‐ GAGGGUGGGGCAUUUCCCUTT‐3’). Cells were harvested 24–48 h posttransfection for Western blot to confirm knockdown efficiency. Cells showing significant SLC2A3 downregulation were used for functional assays.

### 2.13. CCK‐8 Assay and Colony Formation Assay

Posttransfection SW480 cells were seeded in 96‐well plates (2.0 × 10^3^–3.0 × 10^3^ cells/well and *n* = 5 per group). CCK‐8 reagent (10 μL) was added at 0, 24, 48, 72, and 96 h, incubated for 1–2 h, and absorbance measured at 450 nm. Experiments were performed in triplicate, and cell viability was normalized to siNC. Transfected SW480 cells were seeded in 6‐well plates (500–800 cells/well and *n* = 3 per group) and cultured 10–14 days, changing medium every 2–3 days. Colonies (>50 cells) were fixed with 4% paraformaldehyde, stained with 0.1% crystal violet, and counted using ImageJ.

### 2.14. Transwell Invasion Assay

Transwell chambers (8 μm, Corning) coated with diluted Matrigel were used. Transfected SW480 cells (1 × 10^5^ in 200 μL serum‐free medium) were added to the upper chamber; lower chamber contained 600 μL medium with 10% FBS. After 24 h, noninvaded cells were removed, and invaded cells were fixed and stained. Cells were counted in five random fields per well. Experiments were repeated at least three times.

### 2.15. Statistical Analysis

Data preprocessing and analyses were performed in R (v4.5.1), Perl, and GraphPad Prism (v8.0.1). Student’s *t*‐test or Wilcoxon rank‐sum test was used for two‐group comparisons; ANOVA was used for multigroup comparisons. Survival was analyzed with Kaplan–Meier curves, log‐rank tests, and Cox proportional hazards regression. Univariate and multivariate Cox regression analyses estimated hazard ratios (HRs) with 95% CIs. Time‐dependent ROC curves and AUC assessed model predictive performance. *p*‐Values were adjusted using the BH method for FDR. All experiments were performed in triplicate, with significance defined as FDR < 0.05. Data are presented as mean ± SD, with significance levels denoted as  ^∗^ *p* < 0.05,  ^∗∗^ *p* < 0.01, and  ^∗∗∗^ *p* < 0.001.

## 3. Results

### 3.1. Differential Expression and Mutational Burden Characteristics of Parthanatos Gene Signatures

**Figure 1:**
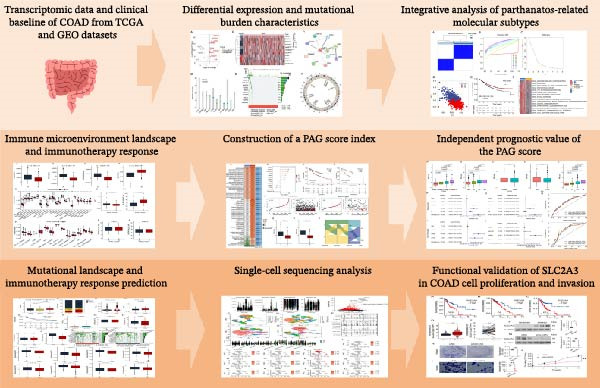
The workflow of data preprocessing.

In this study, a total of 37 PAGs were included to explore their potential regulatory roles in COAD. The overall workflow of the proposed method is illustrated in Figure 1. Differential expression analysis was performed using the criteria of |fold change| ≥ 1.5 and adjusted *p*‐value < 0.05, resulting in the identification of 11 DE‐PAGs (Figure 2A). Heatmap visualization demonstrated that NCF1, ESR1, and ESR2 were significantly upregulated in normal colon tissue samples compared to COAD tumor samples, whereas NAT10, CUL4A, FEN1, and TOMM20 were markedly overexpressed in COAD samples relative to normal controls (Figure 2B). PPI network analysis revealed extensive interactions among the 11 DE‐PAGs, indicating strong functional associations within this gene set (Figure 2C). CNV analysis showed significant amplification of CUL4A and PARP1, while prominent CNV deletions were observed in TOMM20, ESR1, NAMPT, ESR2, GPX4, and FEN1 (Figure 2D). TMB analysis indicated mutation frequencies of 4%, 4%, 3%, 2%, and 2% for ESR1, PARP1, COL8A1, NAT10, and ESR2, respectively (Figure 2E). In addition, chromosomal localization analysis illustrated the genomic distribution of the 11 DE‐PAGs across different chromosomes (Figure 2F). Collectively, these findings systematically characterize the differential expression patterns of PAG in COAD and preliminarily reveal their potential associations with CNVs and somatic mutational burden, providing a basis for subsequent mechanistic investigations.

Figure 2Differential expression analysis and mutational landscape of parthanatos‐associated gene signatures. (A) Differential expression analysis of PAG between normal colon tissues and COAD samples. The screening criteria were set as |fold change| ≥ 1.5 and adjusted *p*‐value < 0.05. Blue dots indicate downregulated genes, whereas red dots indicate upregulated genes. (B) Heatmap visualization of differentially expressed PAG. (C) Protein–protein interaction network analysis of the differentially expressed PAG. (D) Copy number variation frequency analysis of DE‐PAG. (E) Waterfall plot illustrating the somatic mutation frequencies of DE‐PAG. (F) Chromosomal localization analysis showing the distribution of DE‐PAG across different chromosomes.

**Figure:**
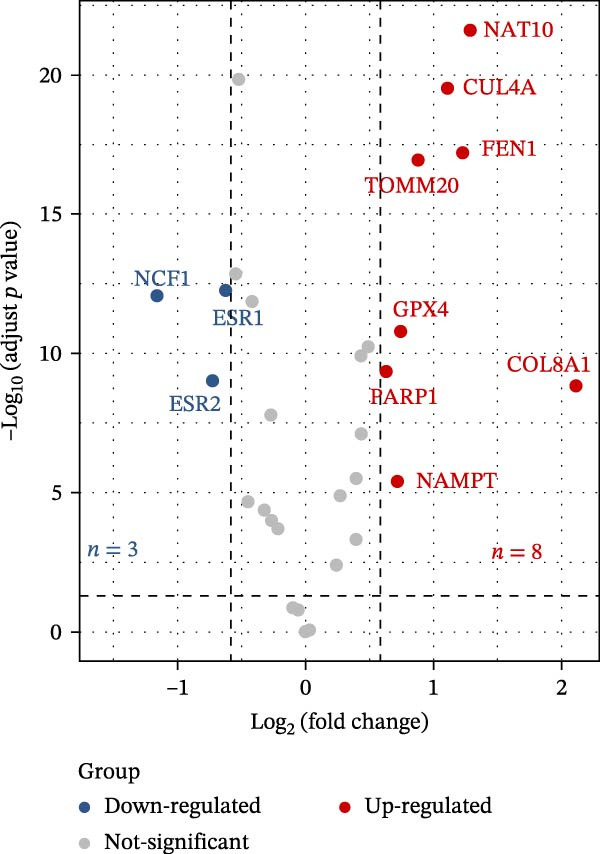
(A)

**Figure:**
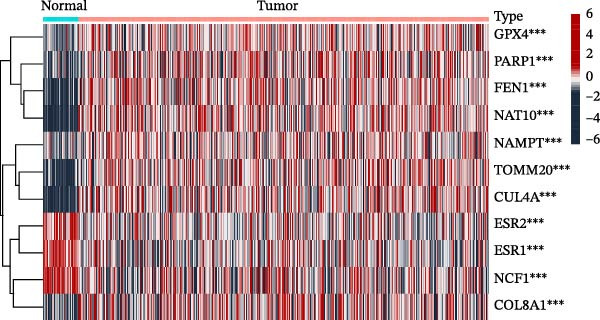
(B)

**Figure:**
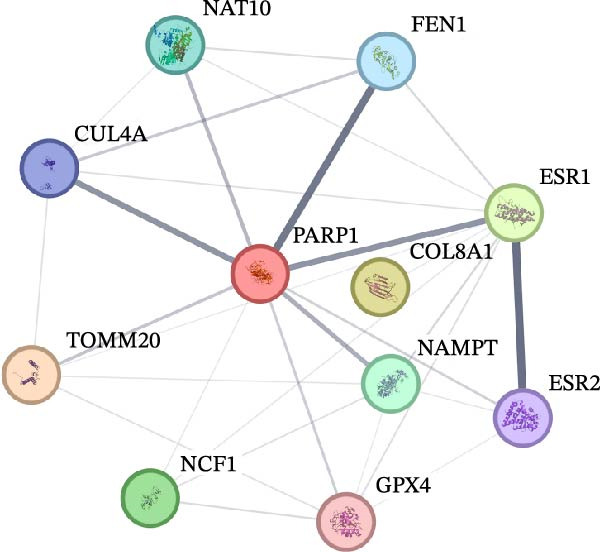
(C)

**Figure:**
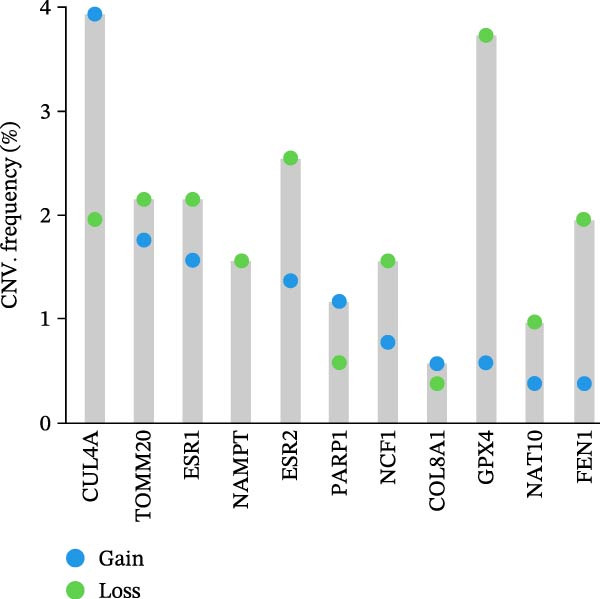
(D)

**Figure:**
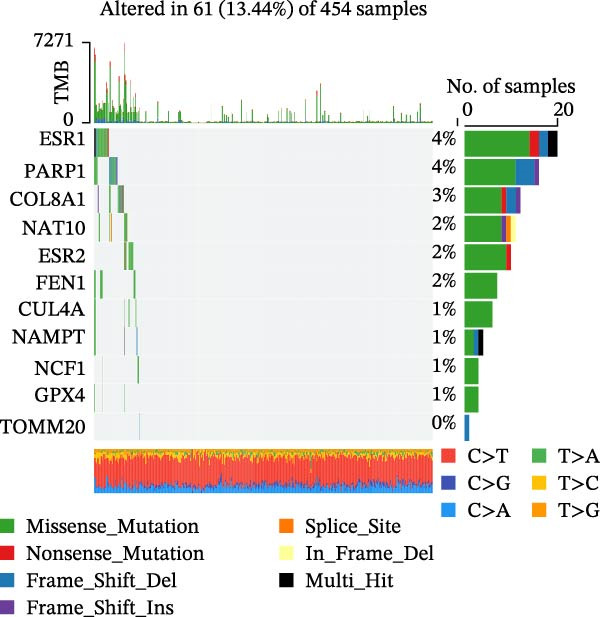
(E)

**Figure:**
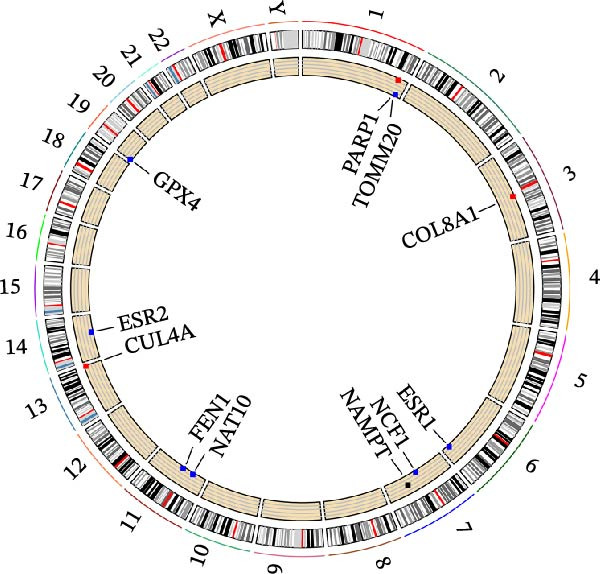
(F)

### 3.2. Integrative Analysis of Parthanatos‐Related Molecular Subtypes and Potential Regulatory Mechanisms

To further investigate the regulatory roles of PAG in COAD, the GSE39582‐COAD and TCGA‐COAD datasets were integrated, yielding a total of 973 COAD samples for molecular subtype analysis. Based on the expression profiles of the 11 DE‐PAGs, unsupervised consensus clustering was performed, and samples were robustly classified into two distinct PAG molecular subtypes: PAG subtype A (441 samples) and PAG subtype B (532 samples) (Figure 3A–C, Supporting Information 2: Figure S1). The PCA plot demonstrated a clear separation between the two PAG subtypes in low‐dimensional space, indicating substantial heterogeneity and independence between them (Figure 3D). Survival analysis revealed that patients in PAG subtype B exhibited significantly better OS than those in subtype A, suggesting a favorable prognostic outcome for subtype B (HR = 1.32 (1.05−1.68) and *p*  = 0.020) (Figure 3E). GSVA based on KEGG pathways revealed pronounced functional differences between the PAG subtypes. In PAG subtype A, the drug metabolism‐other enzymes pathway was significantly downregulated. In contrast, PAG subtype B, which was associated with better prognosis, showed marked downregulation of several tumor progression–related pathways, including pathways in cancer and the TGF‐β signaling pathway. Furthermore, immune‐related pathways such as leukocyte transendothelial migration, cell adhesion molecules (CAMs), and ECM–receptor interaction were also significantly suppressed in subtype B (Figure 3F), suggesting a close relationship between PAG molecular subtypes and the tumor immune microenvironment in COAD.

Figure 3Identification of PAG‐related molecular subtypes and pathway regulation analysis. (A–C) Unsupervised consensus clustering analysis based on differentially expressed parthanatos‐associated gene signatures was performed to identify distinct molecular subtypes in COAD. (D) PCA plot illustrating the separation of PAG molecular subtypes. (E) Kaplan–Meier survival curves based on the Cox test comparing clinical outcomes between different PAG molecular subtypes. (F) GSVA revealing differential regulation of KEGG signaling pathways between PAG molecular subtypes.

**Figure:**
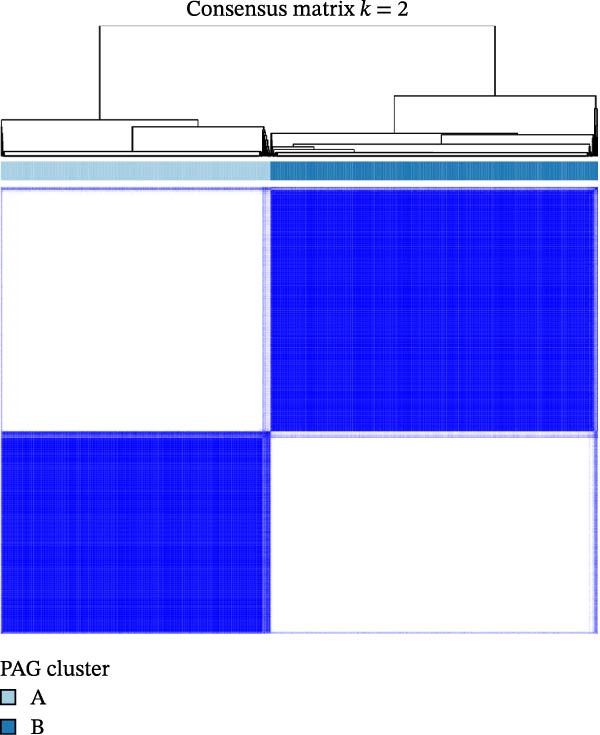
(A)

**Figure:**
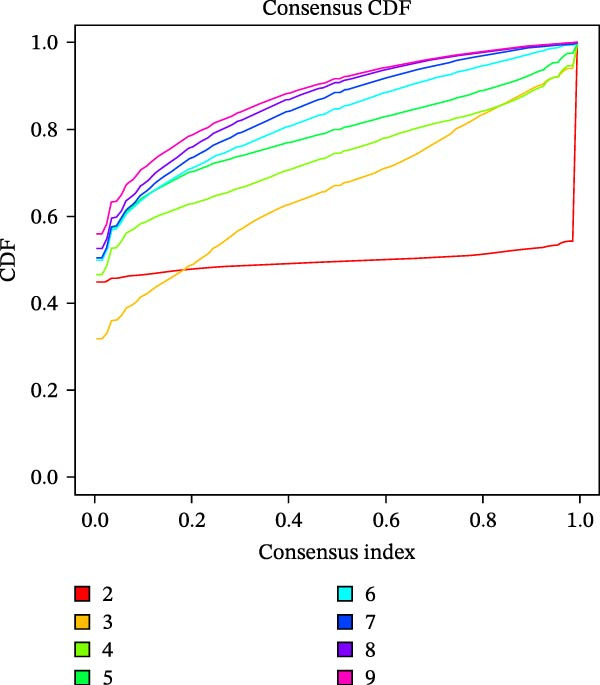
(B)

**Figure:**
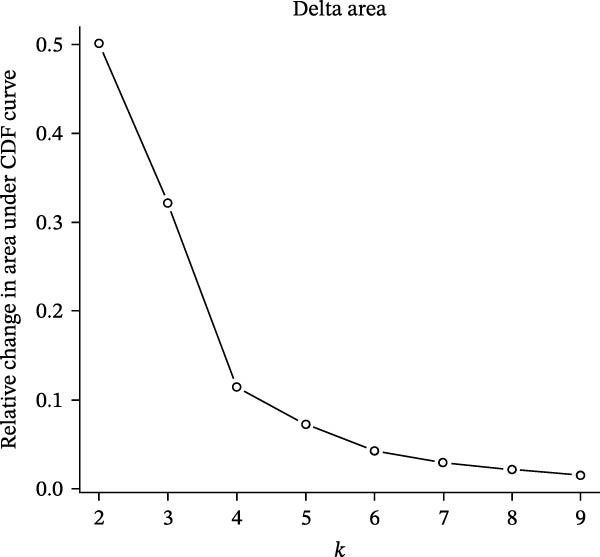
(C)

**Figure:**
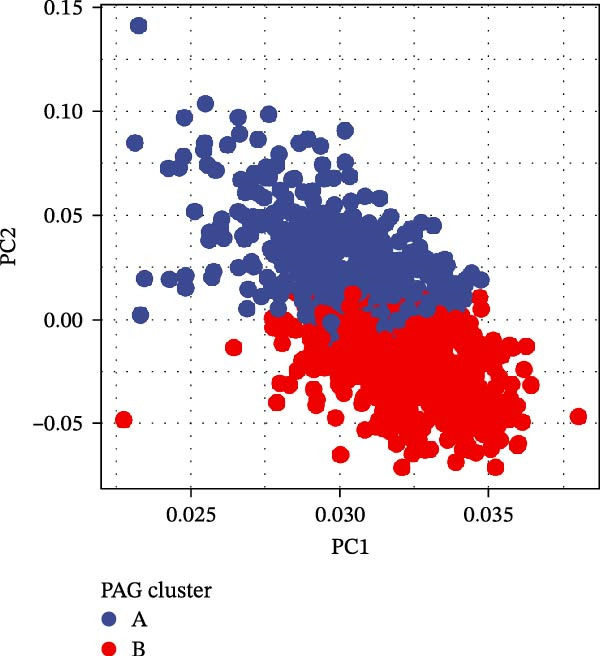
(D)

**Figure:**
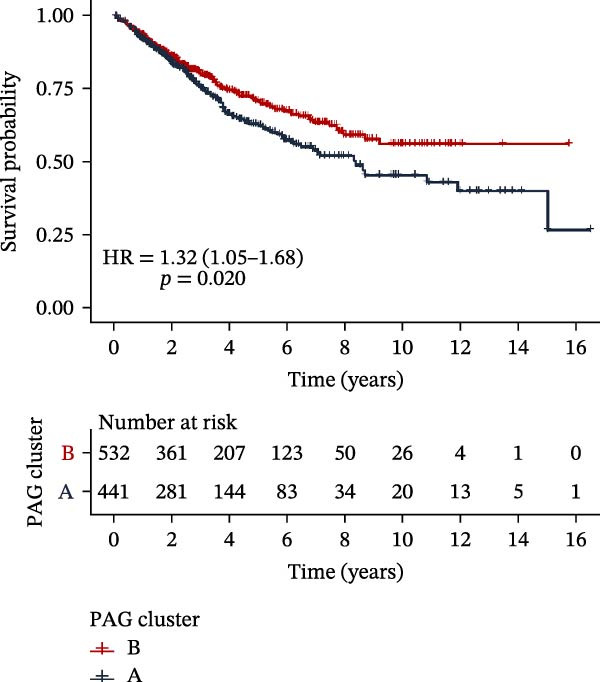
(E)

**Figure:**
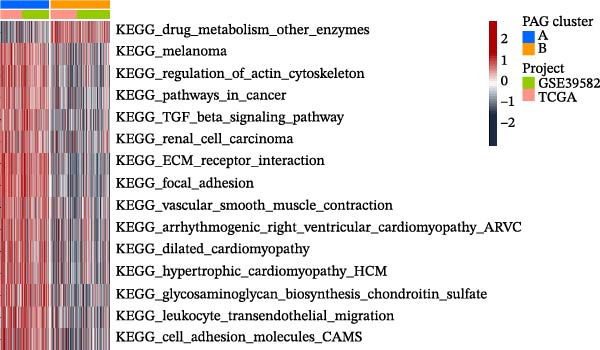
(F)

### 3.3. Immune Microenvironment Landscape and Immunotherapy Response of Parthanatos Molecular Subtypes

Subsequently, multiple immune infiltration algorithms were applied to comprehensively assess differences in the tumor immune microenvironment between PAG subtypes. ESTIMATE analysis showed that PAG subtype B, which exhibited superior clinical outcomes, was characterized by significantly lower immune scores, stromal scores, and ESTIMATE scores, accompanied by increased tumor purity (Figure 4A–D). Using ssGSEA, the infiltration levels of 23 immune cell types were quantified. The results indicated that most immune cell populations, including activated B cells, CD4^+^ T cells, CD8^+^ T cells, and activated dendritic cells, were significantly reduced in PAG subtype B, suggesting an immunosuppressive phenotype (Figure 4E). Further immune functional analysis demonstrated that, compared with PAG subtype A, PAG subtype B showed significantly decreased scores in multiple immune regulatory functions, including APC coinhibition, APC costimulation, CCR, cytolytic activity, and inflammation‐promoting pathways (Figure 4F). Immunotherapy response prediction suggested that PAG subtype B may have a lower likelihood of immune evasion based on TIDE scores, indicating a potentially enhanced response to immune checkpoint inhibitor therapy (Figure 4G). In addition, IPS analysis showed markedly higher scores for subtype B under CTLA‐4–positive and PD‐1–positive conditions, further suggesting a greater likelihood of benefiting from CTLA‐4 or PD‐1–targeted immunotherapy (Figures 4H–J). Taken together, these results highlight the heterogeneity of the immune microenvironment among PAG molecular subtypes and indicate potential differences in immunotherapeutic responsiveness, providing a rationale for precision immunotherapy in COAD.

Figure 4Immune infiltration landscape and immunotherapy response prediction of PAG molecular subtypes. (A–D) Evaluation of the immune infiltration status between different PAG molecular subtypes using the ESTIMATE algorithm. (E) Quantitative assessment of the infiltration levels of 23 immune cell types in PAG molecular subtypes based on the ssGSEA algorithm. (F) Comparison of immune‐related functional scores between PAG molecular subtypes. (G) Differential analysis of TIDE scores between PAG molecular subtypes. (H–J) IPS analysis predicting the potential responses of different PAG molecular subtypes to PD‐1 and/or CTLA‐4 immune checkpoint blockade therapy.

**Figure:**
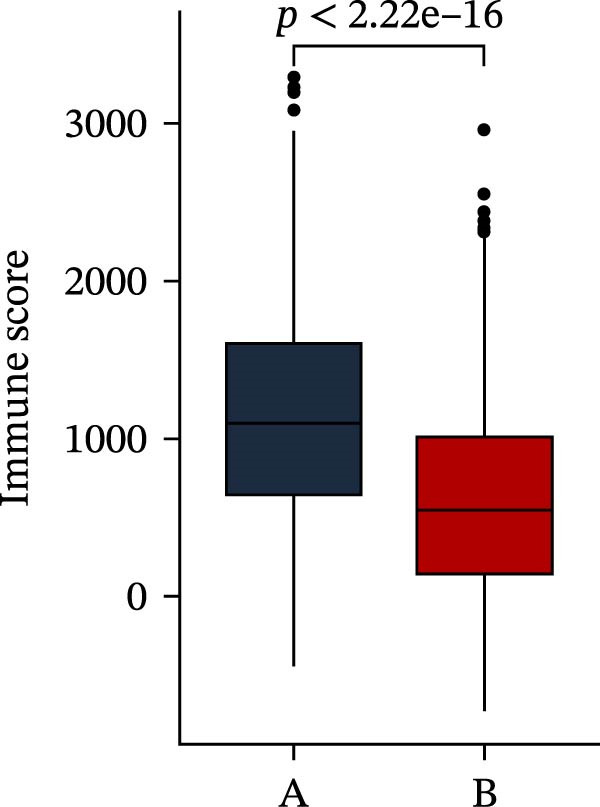
(A)

**Figure:**
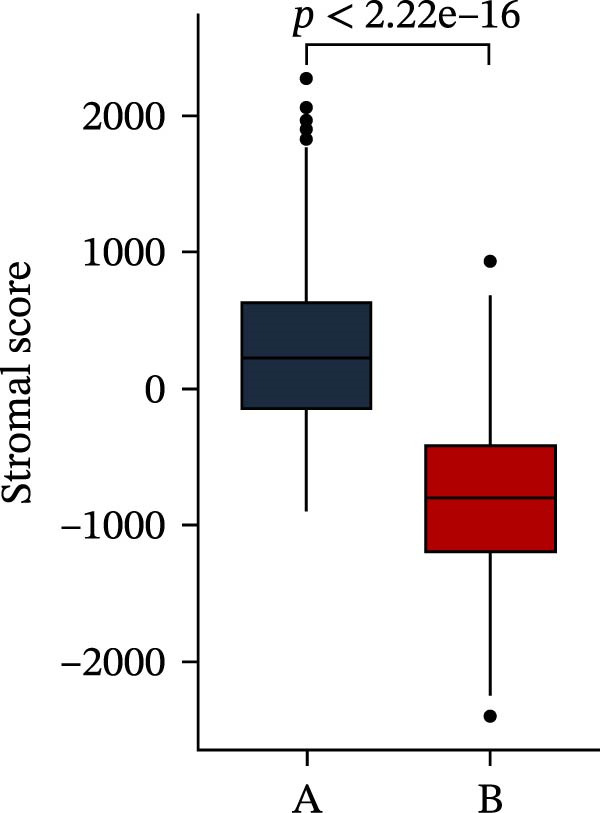
(B)

**Figure:**
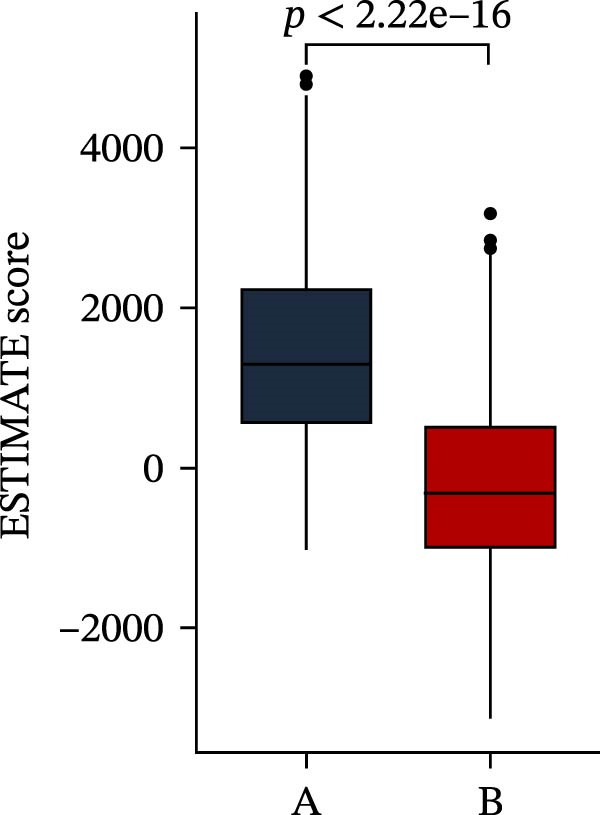
(C)

**Figure:**
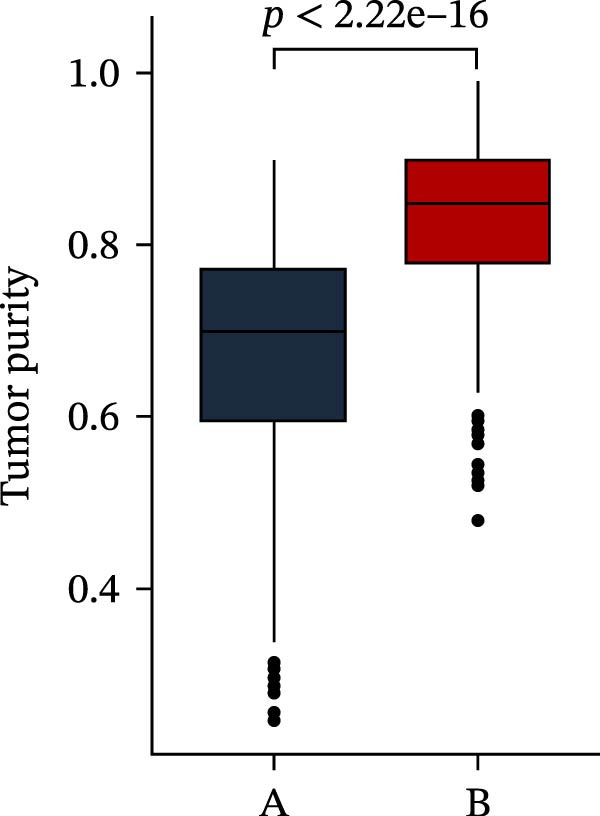
(D)

**Figure:**
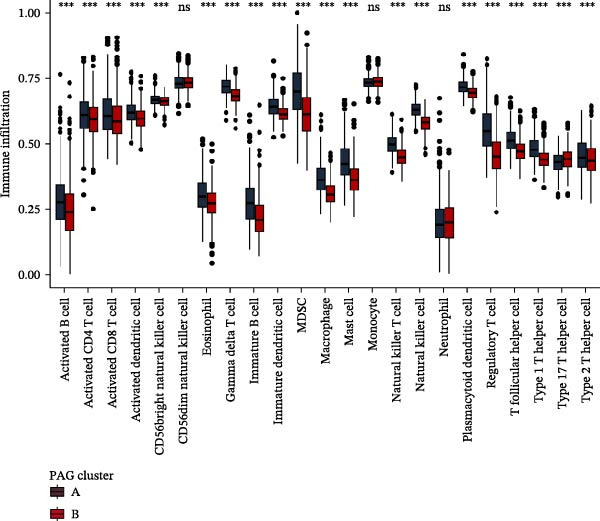
(E)

**Figure:**
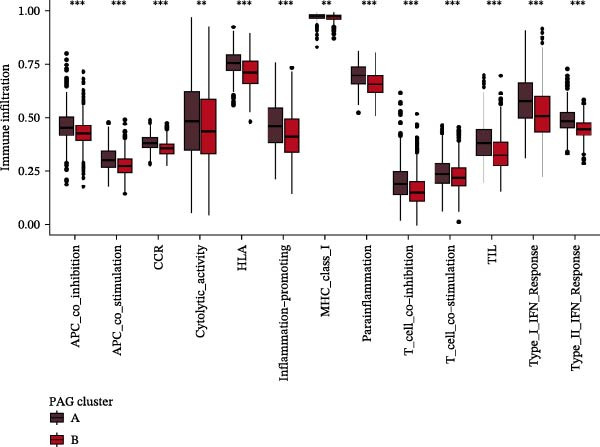
(F)

**Figure:**
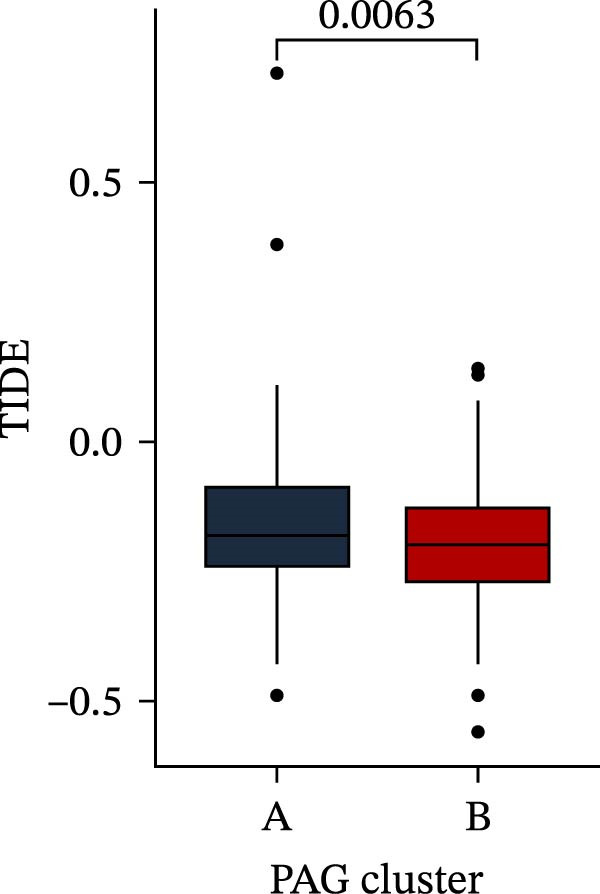
(G)

**Figure:**
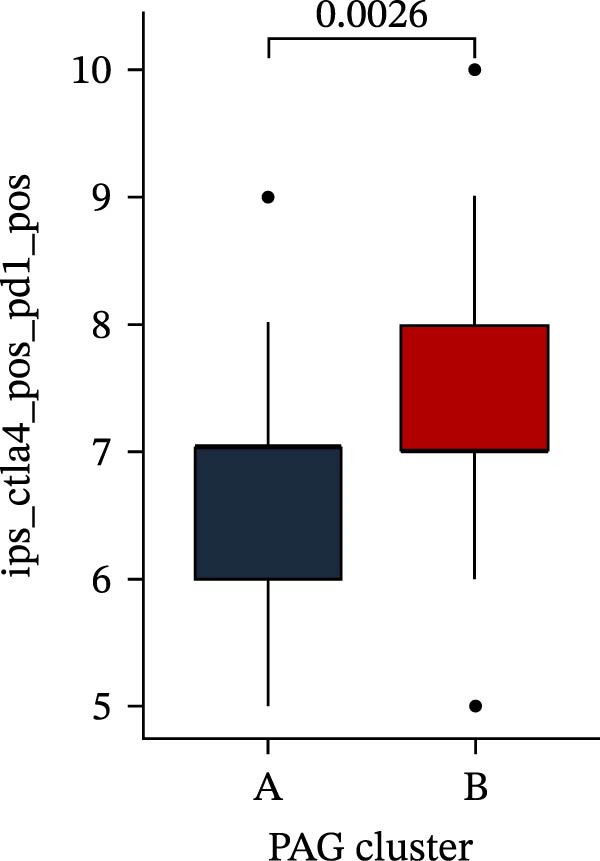
(H)

**Figure:**
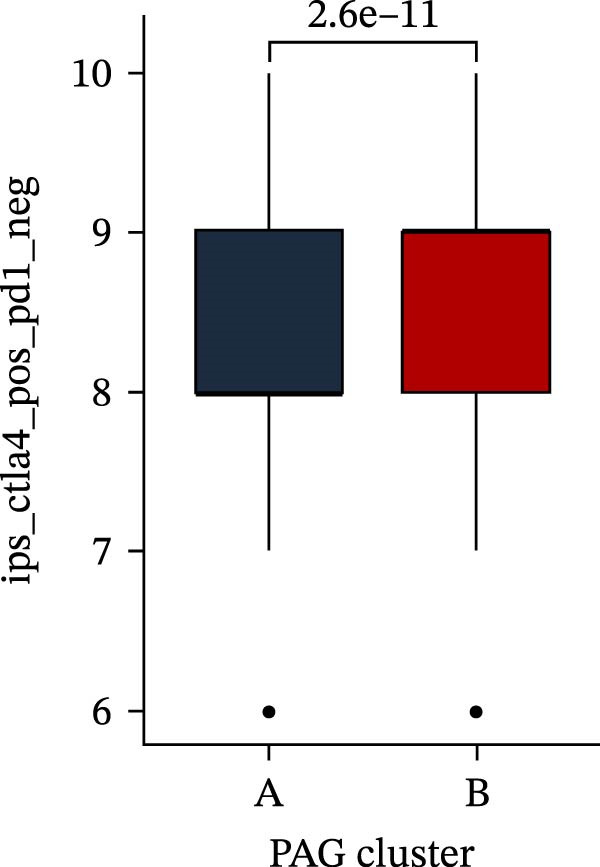
(I)

**Figure:**
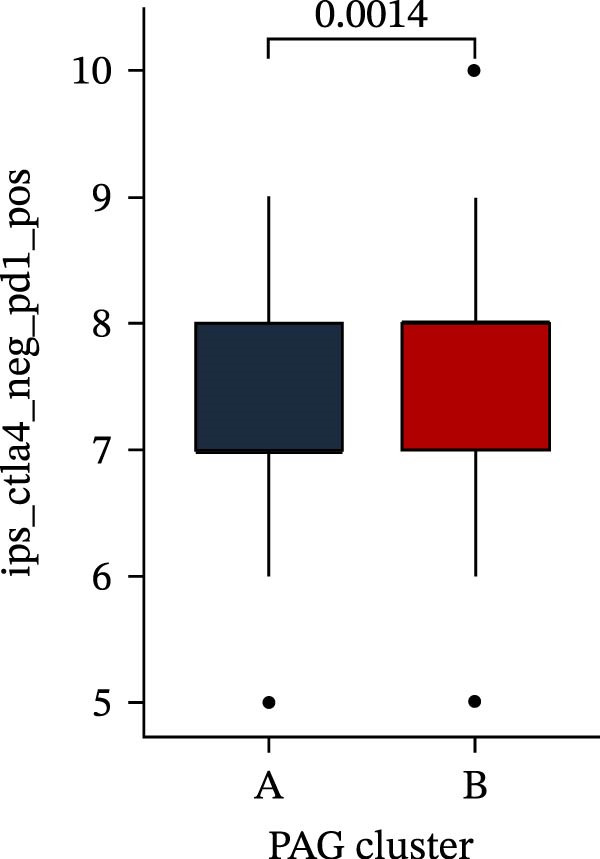
(J)

### 3.4. Identification of PAG Subtypes Associated With Molecular Subtypes

To further elucidate the regulatory mechanisms underlying PAG molecular subtypes, PAG subtype characteristics were systematically analyzed. Using the thresholds of |fold change| ≥ 1.5 and adjusted *p*‐value < 0.05, 871 DEGs were identified between PAG subtypes (Figure 5A). KEGG enrichment analysis indicated that these DEGs were primarily involved in focal adhesion, PI3K–Akt signaling pathway, and cytoskeleton in muscle cells. GO functional enrichment analysis revealed significant enrichment in biological processes related to extracellular matrix organization, extracellular structure organization, collagen‐containing extracellular matrix, and extracellular matrix structural constituent (Figure 5B, C). Based on the expression profiles of these DEGs, unsupervised consensus clustering further classified COAD samples into two gene subtypes: gene subtype A (328 samples) and gene subtype B (645 samples) (Figure 5D–F). PCA confirmed clear separation between the two gene subtypes, supporting their distinct biological identities (Figure 5G). Survival analysis demonstrated that patients in gene subtype B had a significantly better prognosis than those in subtype A (HR = 1.52 (1.20−1.94) and *p*  < 0.001) (Figure 5H). Heatmap visualization further illustrated DEG expression patterns across clinical features and molecular subtypes, with the majority of DEGs markedly upregulated in gene subtype A (Figure 5I).

Figure 5Identification of gene subtypes associated with PAG molecular subtypes. (A) Identification of differentially expressed genes between PAG molecular subtypes. The thresholds were set as |fold change| ≥ 1.5 and adjusted *p*‐value < 0.05. Red dots represent upregulated genes, whereas blue dots represent downregulated genes. (B, C) KEGG and GO enrichment analyses of the DEGs. (D–F) Unsupervised consensus clustering based on DEGs to identify distinct gene subtypes. (G) PCA plot showing the separation patterns between gene subtypes. (H) Kaplan–Meier survival curves of gene subtypes based on the Cox test. (I) Heatmap illustrating the expression levels of DEGs across different clinicopathological features and molecular subgroups.

**Figure:**
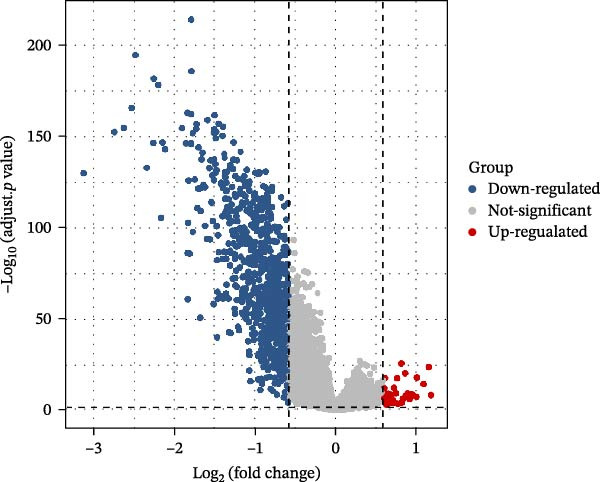
(A)

**Figure:**
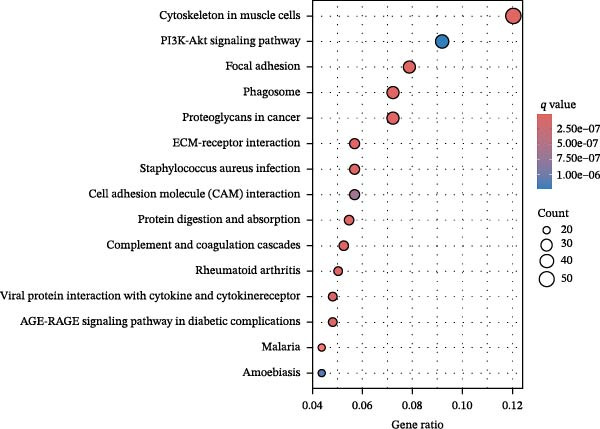
(B)

**Figure:**
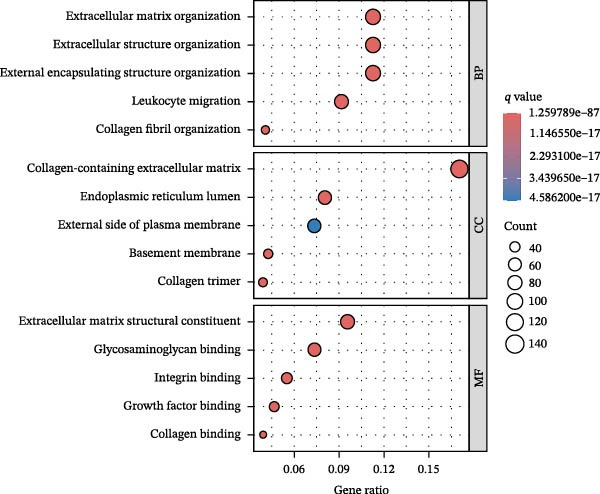
(C)

**Figure:**
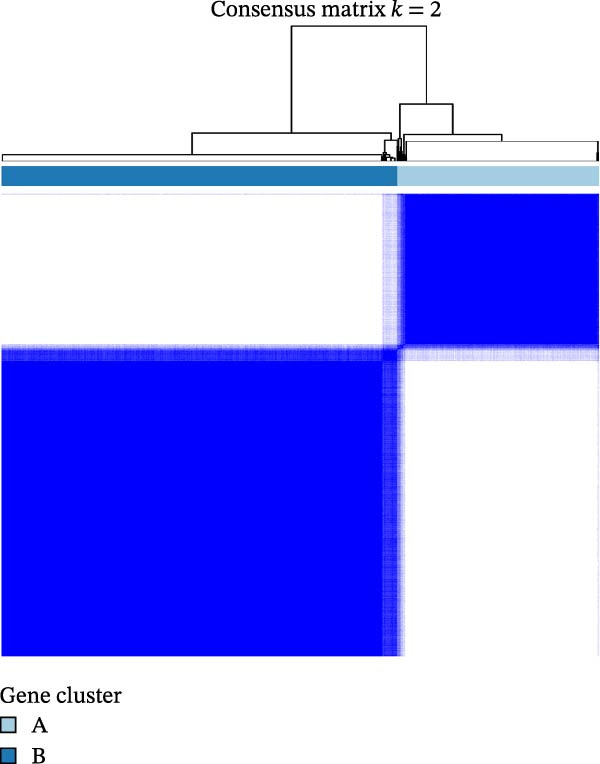
(D)

**Figure:**
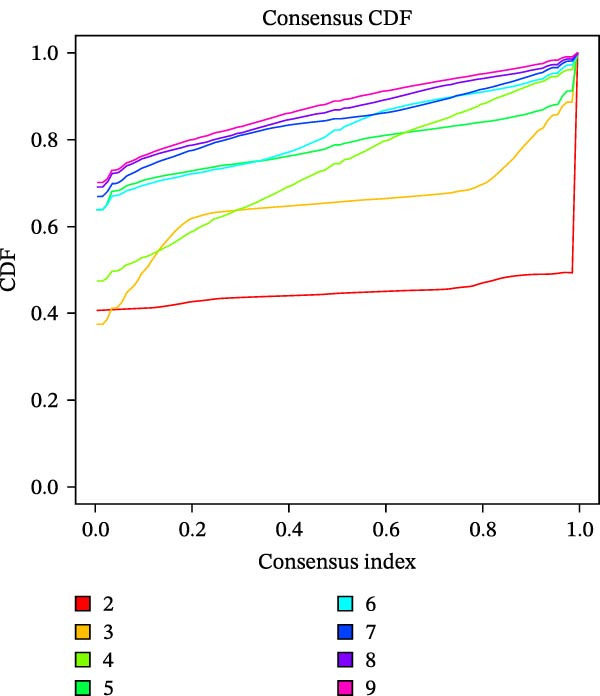
(E)

**Figure:**
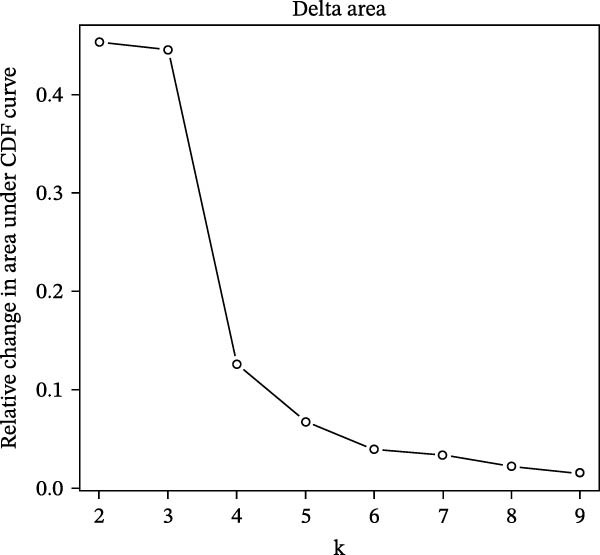
(F)

**Figure:**
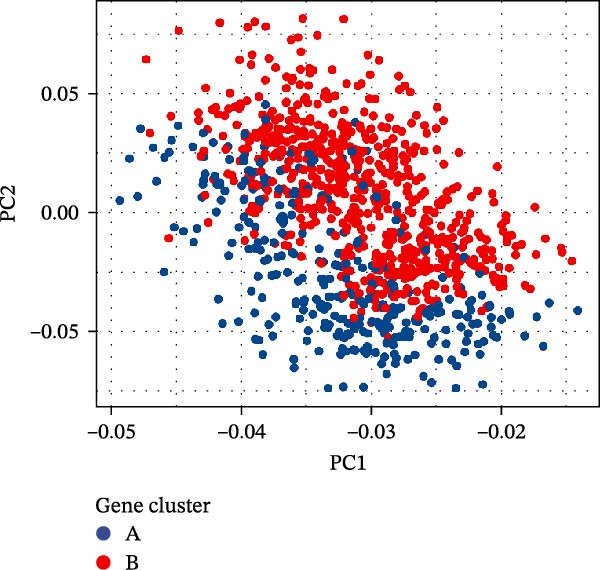
(G)

**Figure:**
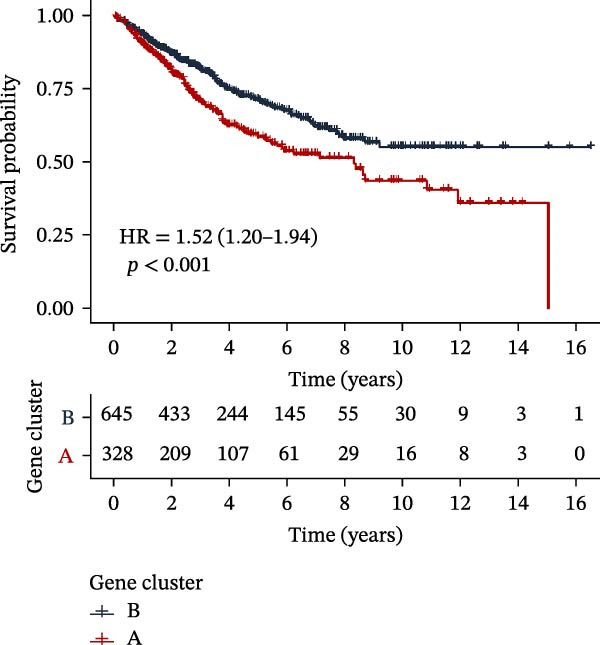
(H)

**Figure:**
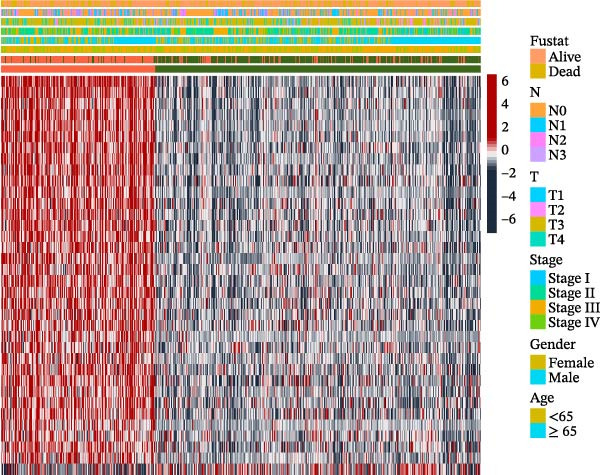
(I)

### 3.5. Construction of a PAG Score Index Using Integrated Machine Learning Algorithms for Prognostic Prediction

To construct a PAG score capable of predicting clinical outcomes in COAD, multiple machine learning algorithms were integrated to perform risk stratification in both training and external validation cohorts. The GSE17538‐COAD dataset was used as an independent external validation cohort. Clinical survival information and DEG expression profiles from the training cohort were integrated for model development. Within a LOOCV framework, 64 combinations derived from 10 machine learning algorithms were evaluated, and C‐index values were calculated in both training and validation cohorts. The Elastic Net regression model (Enet [alpha = 0.1] and C‐index = 0.665) demonstrated the best performance and was subsequently used to select key prognostic variables (Figure 6A). Multivariate Cox regression analysis identified seven variables that were significantly associated with unfavorable prognosis in COAD, among which SLC2A3 exhibited the highest risk coefficient (Figure 6B). Based on the regression coefficients and expression levels of these prognostic variables, a PAG score was calculated for each sample in both the training cohort and the GSE17538 validation cohort. Samples were subsequently classified into high and low PAG score subgroups using the median value as the cutoff. Kaplan–Meier survival analysis demonstrated that patients in the low PAG score subgroup consistently exhibited significantly better clinical outcomes than those in the high PAG score subgroup in both the training and validation cohorts (Figure 6C–F). Comparative analysis further revealed that PAG subtype B and gene subtype B, both associated with favorable prognosis, exhibited significantly lower PAG scores (Figure 6G, H). Sankey diagram analysis illustrated the potential associations among molecular subtypes, PAG score subgroups, and clinical survival outcomes in COAD (Figure 6I). Overall, these results demonstrate that the PAG score is a stable and reliable prognostic indicator capable of effective risk stratification in COAD and is closely associated with molecular subtypes and clinical outcomes, with higher PAG scores indicating poorer prognosis.

Figure 6Development and validation of the PAG score model integrating multiple machine learning algorithms. (A) Calculation of the C‐index in the training and validation cohorts by integrating 64 algorithm combinations derived from 10 machine learning methods under the LOOCV framework. (B) Distribution of risk coefficients for independently prognostic variables. (C, D) Kaplan–Meier survival curves based on the log‐rank test in the training cohort and the GSE17538 validation cohort. (E, F) Stratification of patients into PAG score subgroups in the training cohort and the GSE17538 validation cohort. (G, H) Differential analysis of PAG scores across PAG molecular subtypes and gene subtypes. (I) Sankey diagram illustrating the potential associations among PAG molecular subtypes, gene subtypes, PAG score groups, and clinical outcomes.

**Figure:**
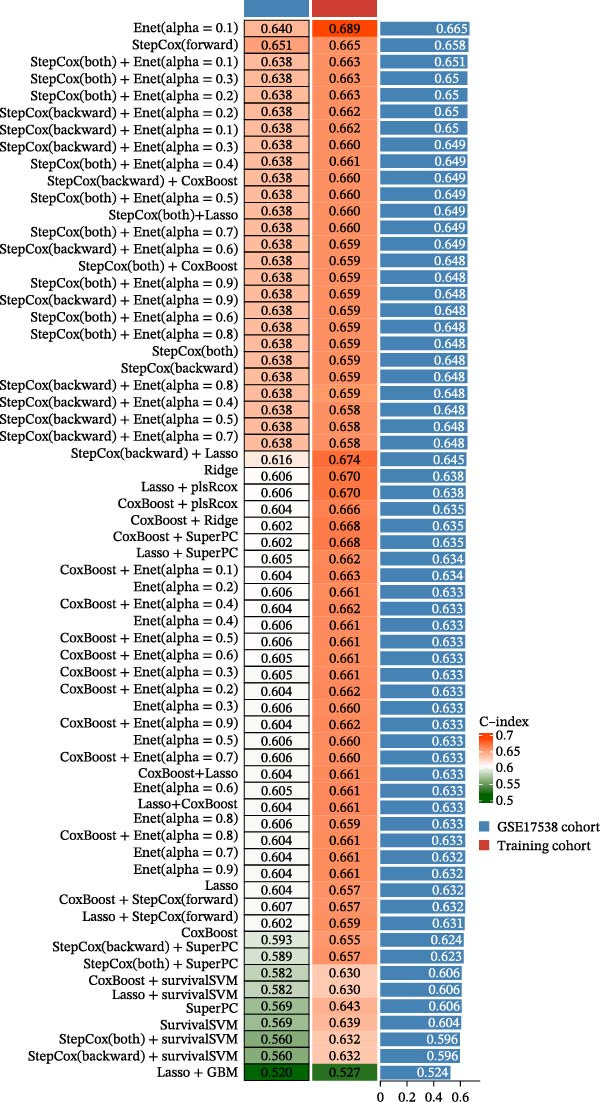
(A)

**Figure:**
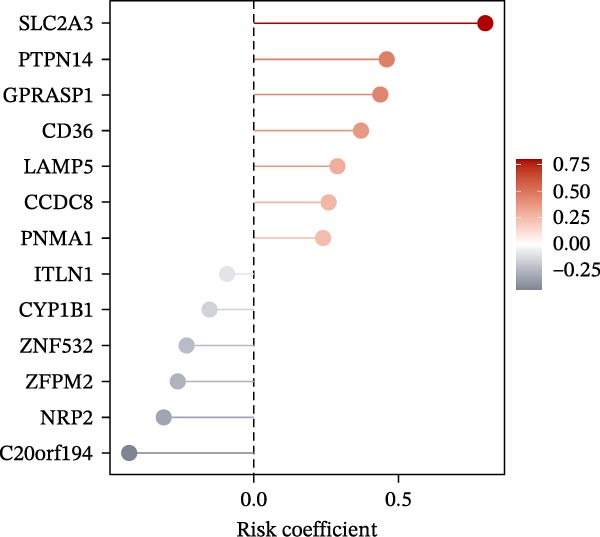
(B)

**Figure:**
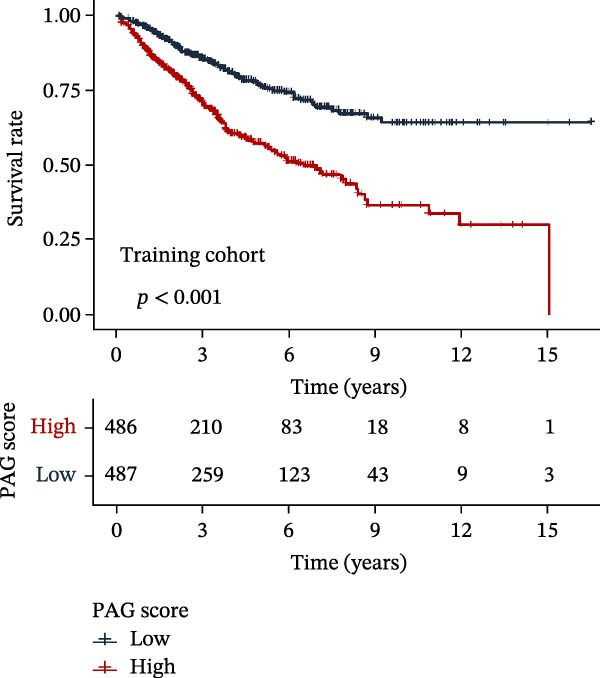
(C)

**Figure:**
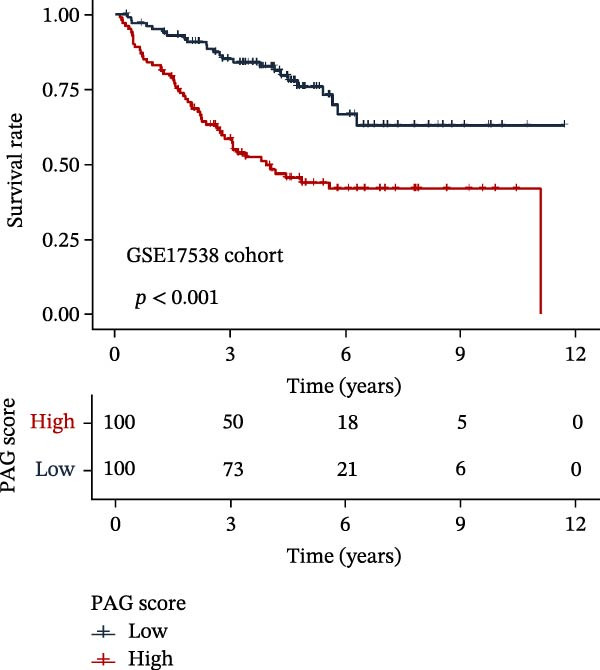
(D)

**Figure:**
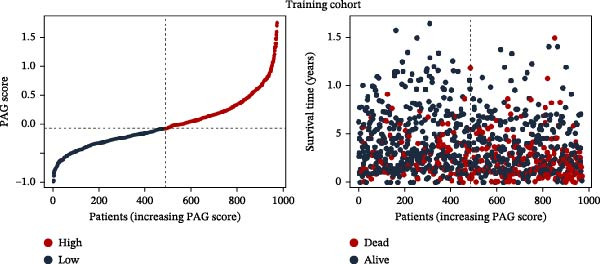
(E)

**Figure:**
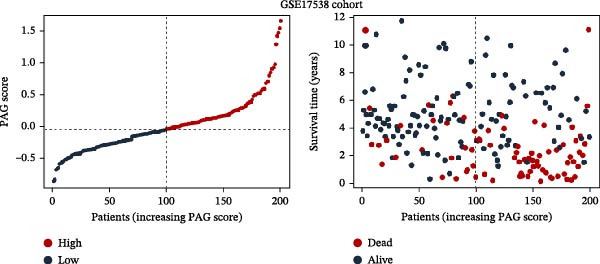
(F)

**Figure:**
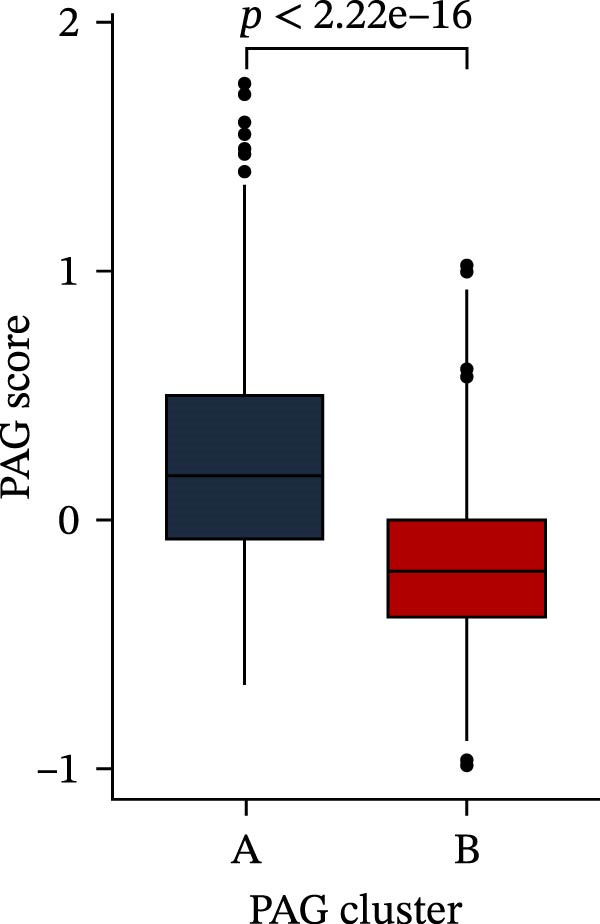
(G)

**Figure:**
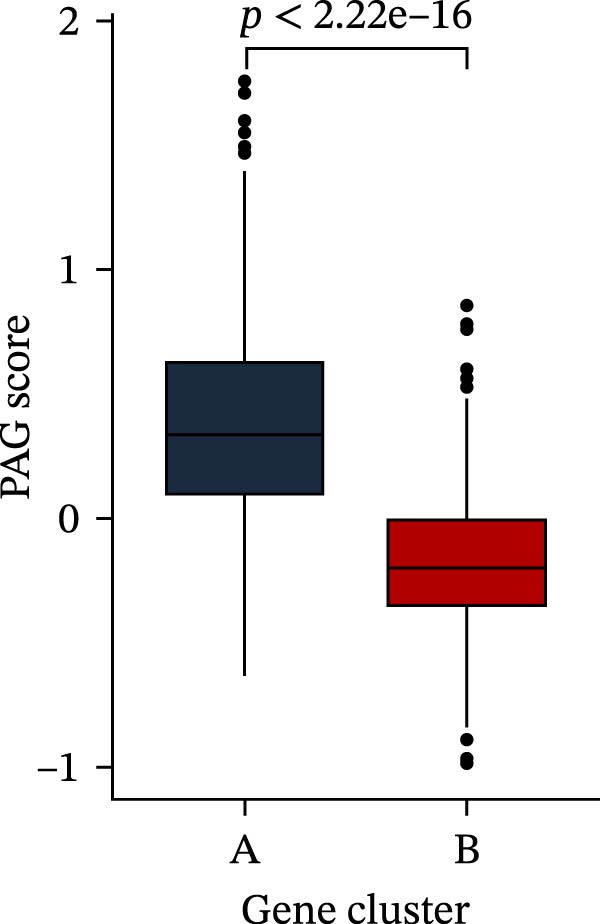
(H)

**Figure:**
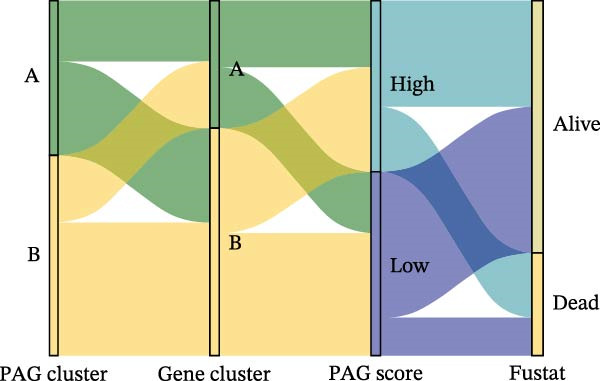
(I)

### 3.6. Distribution and Independent Prognostic Value of the PAG Score in Clinicopathological Subgroups

To further evaluate the distribution patterns and independent prognostic value of the PAG score across clinicopathological subgroups, systematic analyses were conducted in both the training and validation cohorts. Subgroup analysis revealed that PAG scores were significantly elevated in patients with advanced tumor stage, N stage, and T stage, whereas no significant differences were observed across age or sex subgroups (Figure 7A–E). In the training cohort, univariate Cox regression analysis showed that stage (HR = 2.196, 95% CI: 1.828–2.639, and *p*  < 0.001), T stage (HR = 2.076, 95% CI: 1.613–2.674, and *p*  < 0.001), N stage (HR = 1.536, 95% CI: 1.313–1.797, and *p*  < 0.001), and the PAG score (HR = 2.792, 95% CI: 2.054–3.796, and *p*  < 0.001) were all significantly associated with poor prognosis (Figure 7F). Multivariate Cox regression analysis further identified stage (HR = 2.179, 95% CI: 1.699–2.796, and *p*  < 0.001), T stage (HR = 1.601, 95% CI: 1.203–2.130, and *p* = 0.001), and the PAG score (HR = 2.224, 95% CI: 1.605–3.081, and *p*  < 0.001) as independent prognostic factors (Figure 7G). Time‐dependent ROC analysis demonstrated that the PAG score achieved an AUC of 0.682, 0.660, and 0.660 for predicting 1‐, 3‐, and 5‐year OS, respectively, in the training cohort (Figure 7H). In the validation cohort, univariate Cox regression analysis showed that stage, grade, and the PAG score were significantly associated with poor prognosis (Figure 7I), while multivariate analysis confirmed stage and the PAG score as independent prognostic factors (Figure 7J). The AUC of the PAG score for predicting 1‐, 3‐, and 5‐year OS in the validation cohort was 0.722, 0.733, and 0.720, respectively (Figure 7K). These findings indicate that the PAG score serves as a robust and independent prognostic indicator beyond traditional clinicopathological characteristics in COAD.

Figure 7Analysis of PAG score distribution across clinicopathological subgroups and its independent prognostic value. (A–E) Differential analysis of PAG scores across different clinicopathological characteristics, including stage, N stage, gender, age, and T stage. (F, G) Univariate and multivariate Cox regression analyses of clinicopathological variables and the PAG score in the training cohort. (H) Time‐dependent ROC curve analysis for predicting 1‐, 3‐, and 5‐year overall survival in the training cohort. (I, J) Univariate and multivariate Cox regression analyses of clinicopathological variables and the PAG score in the GSE17538 validation cohort. (K) Time‐dependent ROC curve analysis for predicting 1‐, 3‐, and 5‐year overall survival in the GSE17538 validation cohort.

**Figure:**
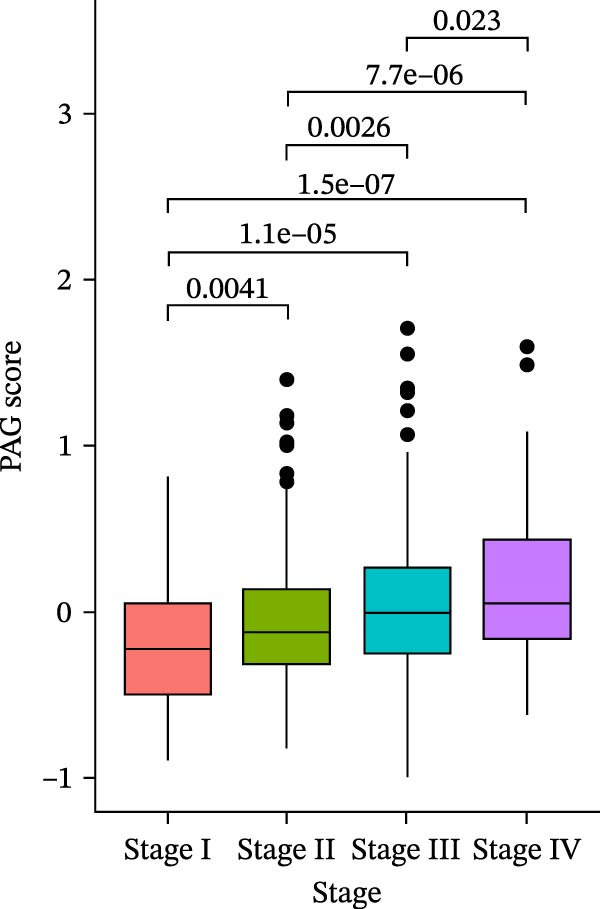
(A)

**Figure:**
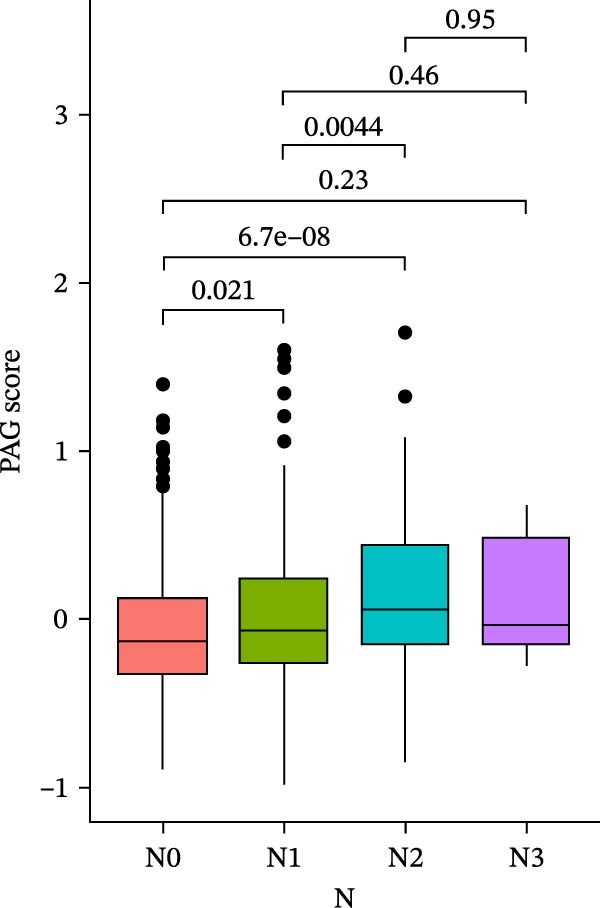
(B)

**Figure:**
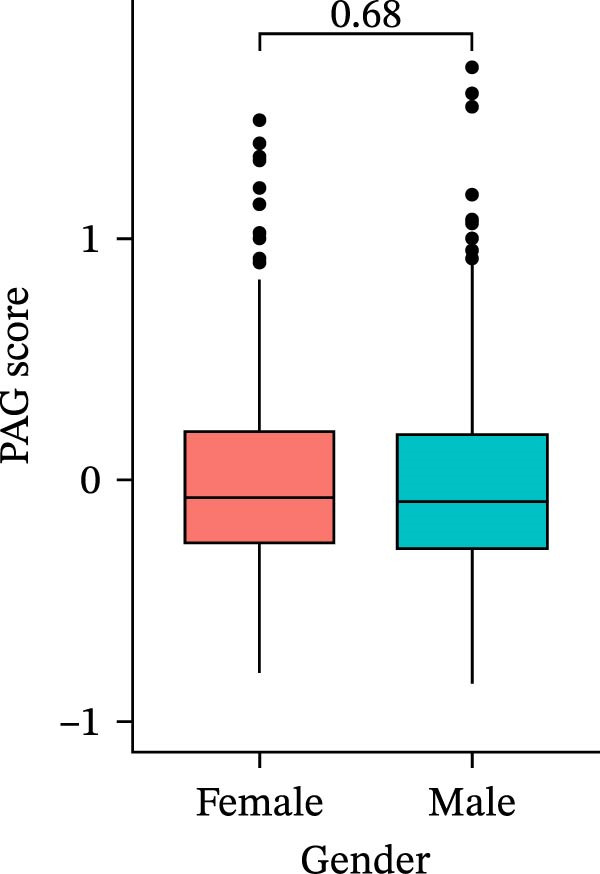
(C)

**Figure:**
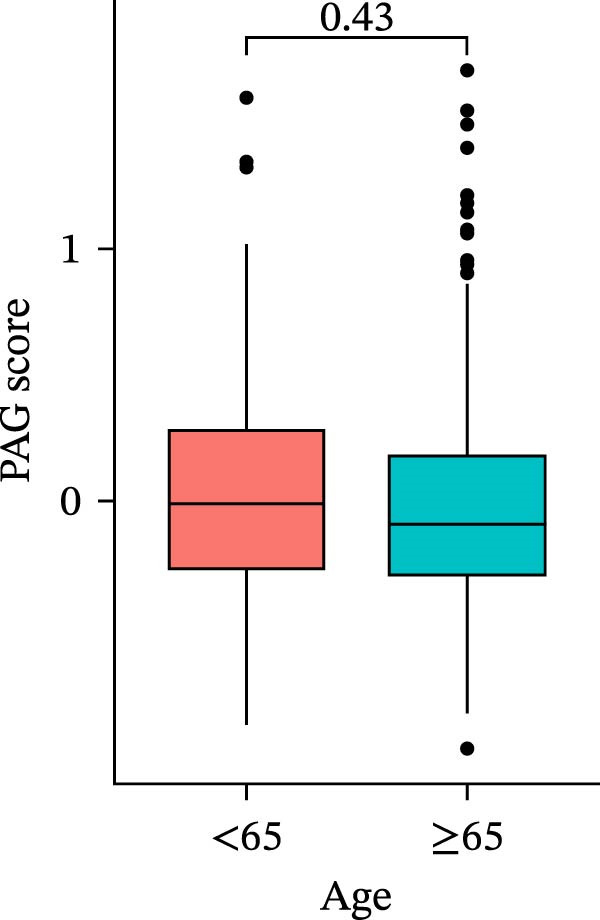
(D)

**Figure:**
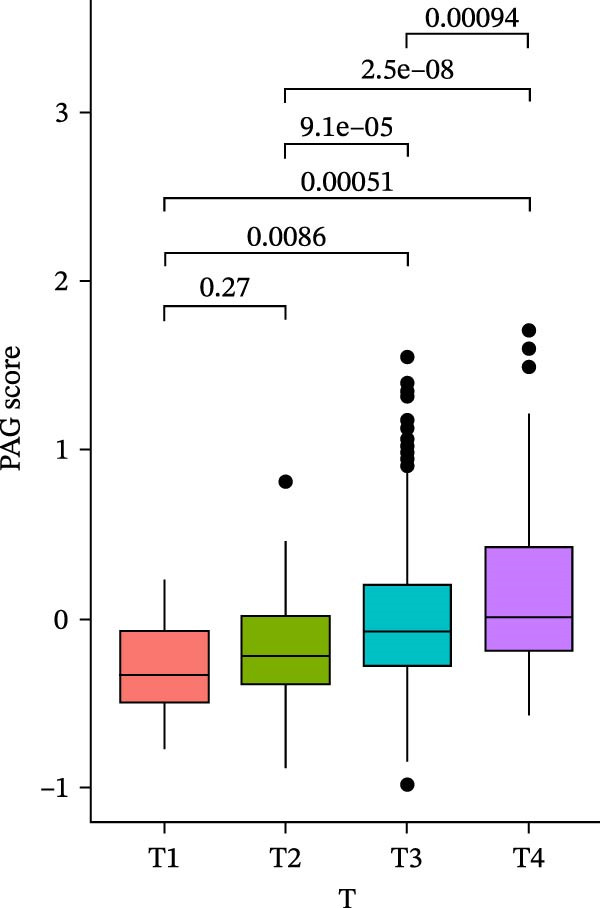
(E)

**Figure:**
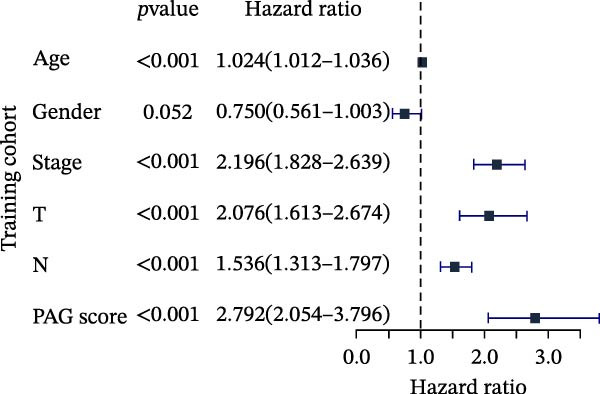
(F)

**Figure:**
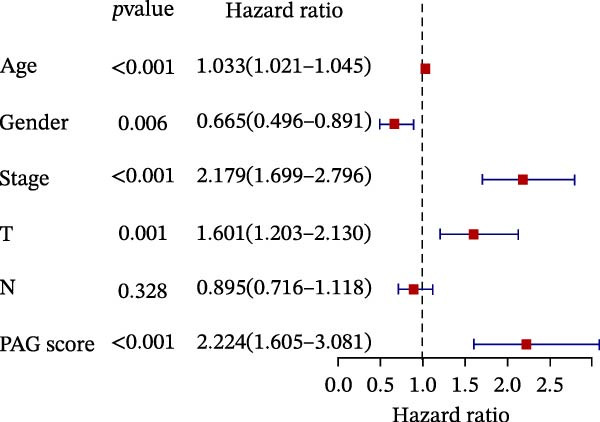
(G)

**Figure:**
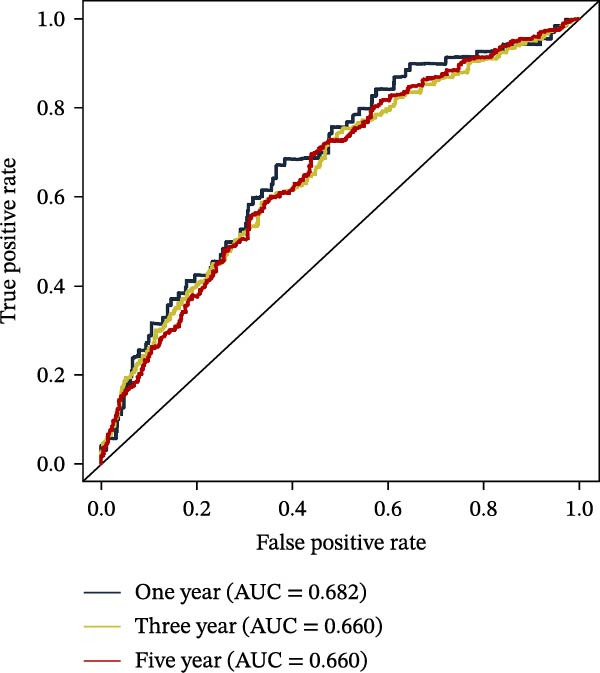
(H)

**Figure:**
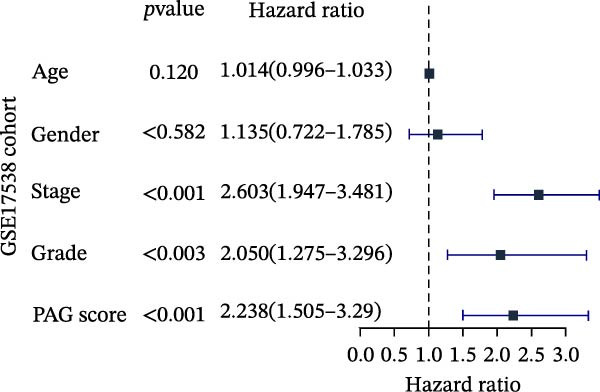
(I)

**Figure:**
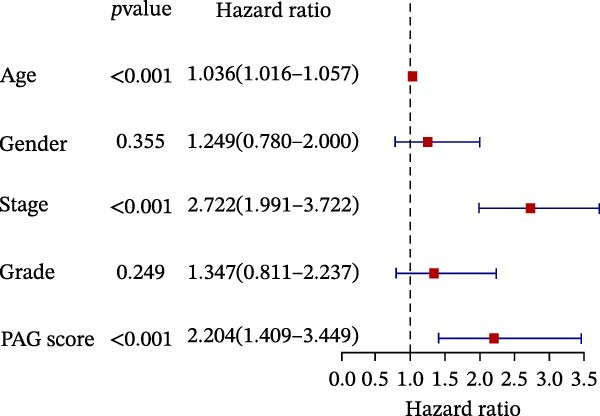
(J)

**Figure:**
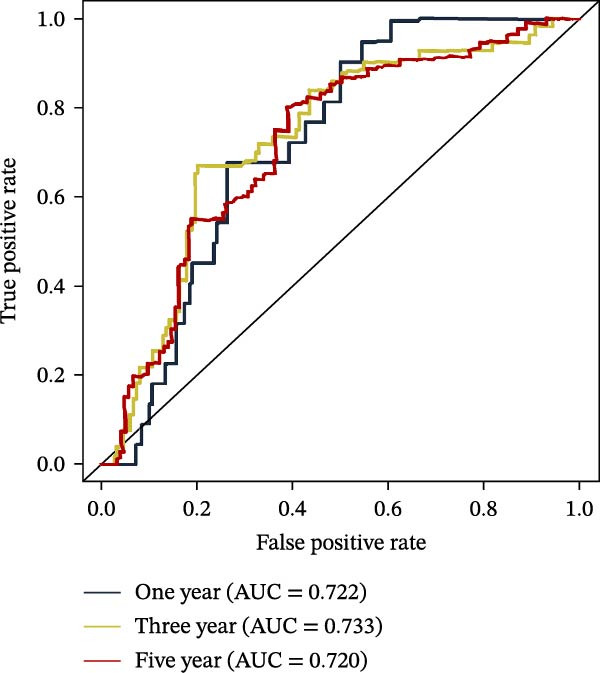
(K)

### 3.7. Development of a Clinicopathology‐Based Nomogram for Survival Prediction

Based on clinicopathological variables and the PAG score, nomogram models were constructed in both the training and validation cohorts to predict 1‐, 3‐, and 5‐year survival probabilities. In both cohorts, the nomograms integrating clinicopathological factors and the PAG score accurately predicted survival outcomes at different follow‐up time points (Figure 8A, B). Calibration curve analysis demonstrated a high concordance between predicted and observed survival probabilities, indicating good calibration and stability of the nomogram models (Figure 8C, E). Furthermore, C‐index analysis confirmed the predictive accuracy and reliability of individual clinicopathological variables and the PAG score for estimating clinical survival outcomes in COAD (Figure 8D, F). Overall, these nomogram models effectively integrate the PAG score with traditional clinicopathological factors, providing a quantitative, visual, and clinically applicable tool for prognostic evaluation in COAD.

Figure 8Development and calibration of a nomogram prediction model based on clinicopathological variables and the PAG score. (A, B) Construction of nomogram models integrating clinicopathological variables and the PAG score in the training cohort and the GSE17538 validation cohort. (C) Calibration curve analysis of the nomogram model in the training cohort. The diagonal represents the ideal forecast. (D) C‐index analysis of clinicopathological variables and the PAG score in the training cohort. (E) Calibration curve analysis of the nomogram model in the GSE17538 validation cohort. The diagonal represents the ideal forecast. (F) C‐index analysis of clinicopathological variables and the PAG score in the GSE17538 validation cohort.

**Figure:**
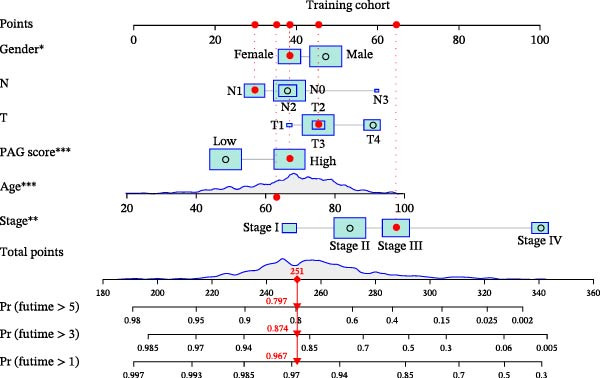
(A)

**Figure:**
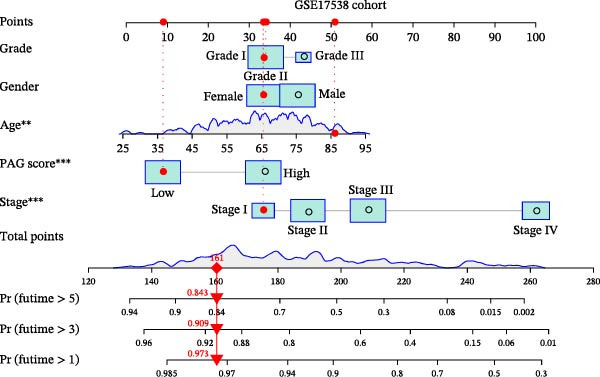
(B)

**Figure:**
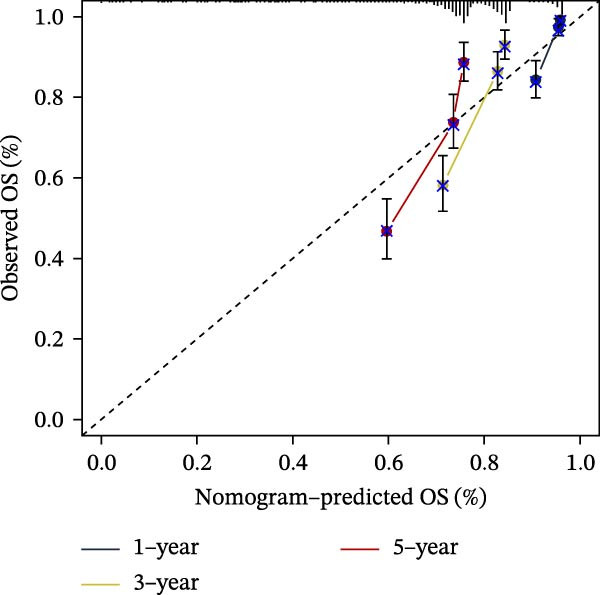
(C)

**Figure:**
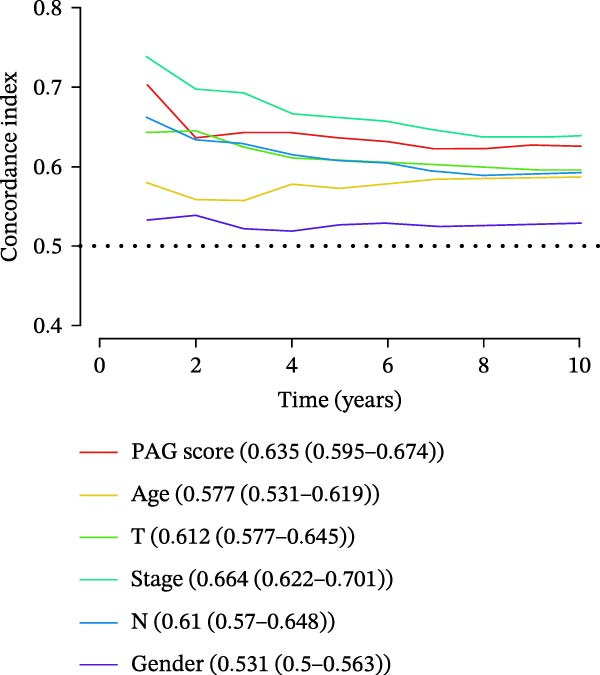
(D)

**Figure:**
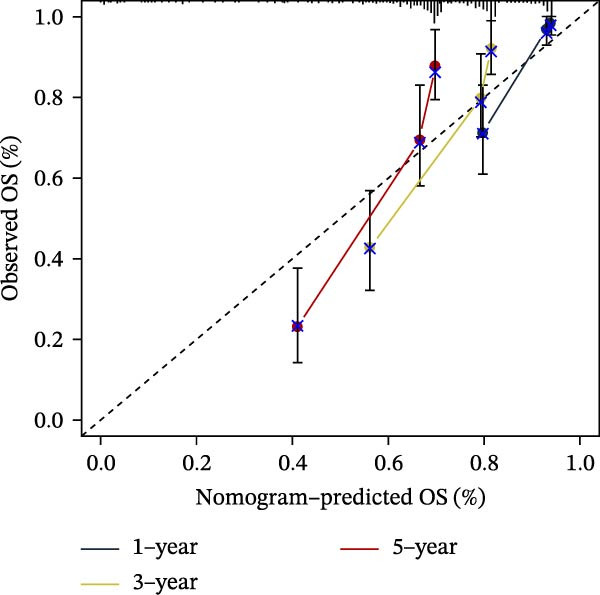
(E)

**Figure:**
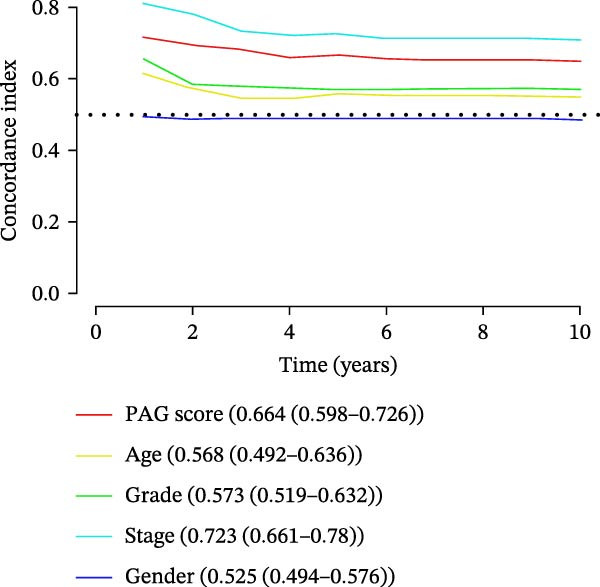
(F)

### 3.8. Immune Microenvironment Landscape and Enrichment Analysis of PAG Score Subgroups

To further elucidate the molecular regulatory mechanisms underlying different PAG score subgroups, differential expression analysis was first performed between the high and low PAG score groups to identify DEGs. GO enrichment analysis indicated that these DEGs were primarily involved in chemotaxis, immune receptor activity, and extracellular matrix organization, while KEGG pathway analysis revealed enrichment in cytoskeleton in muscle cells, chemokine signaling pathway, CAM interaction, and ECM–receptor interaction (Figure 9A, B). ESTIMATE analysis demonstrated that, compared with the low PAG score subgroup, the high PAG score subgroup exhibited significantly lower tumor purity and markedly higher stromal, immune, and ESTIMATE scores, indicating a more complex tumor microenvironment (Figure 9C–F). Immune functional analysis revealed that APC costimulation, type I interferon (IFN) response, and type II IFN response scores were significantly elevated in the high PAG score subgroup, whereas T cell costimulation scores were significantly reduced (Figure 9G). ssGSEA‐based immune cell infiltration analysis showed that the proportions of activated B cells, CD8^+^ T cells, monocytes, neutrophils, and type 17 T helper cells were significantly decreased in the high PAG score subgroup, while gamma delta T cells, MDSCs, macrophages, and natural killer (NK) T cells were significantly increased (Figure 9H). These results demonstrate substantial differences in immune microenvironment composition and immune regulatory patterns between PAG score subgroups.

Figure 9Immune microenvironment landscape and enrichment analysis of PAG score subgroups. (A, B) GO and KEGG enrichment analyses of differentially expressed genes between PAG score subgroups. (C–F) Evaluation of immune infiltration status using the ESTIMATE algorithm. (G) Immune function scoring assessment. (H) Quantitative analysis of the infiltration proportions of 23 immune cell types in PAG score subgroups based on the ssGSEA algorithm.

**Figure:**
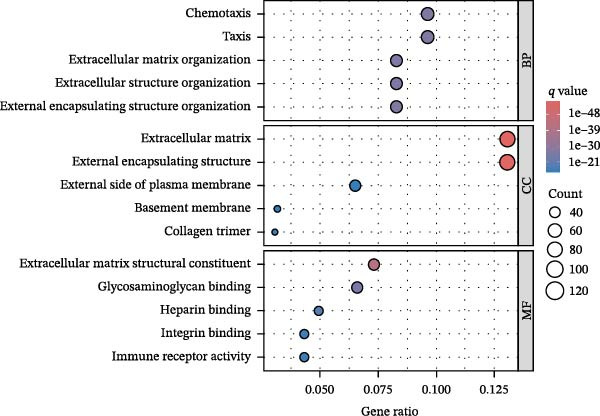
(A)

**Figure:**
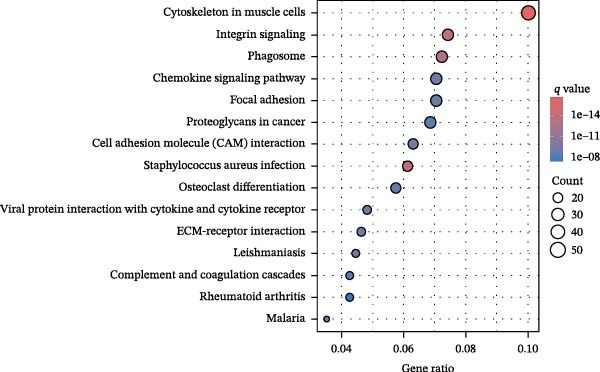
(B)

**Figure:**
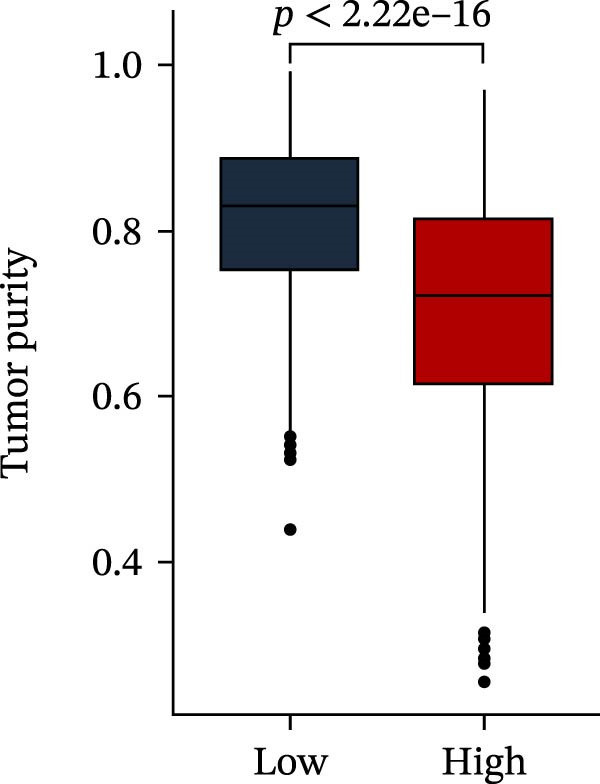
(C)

**Figure:**
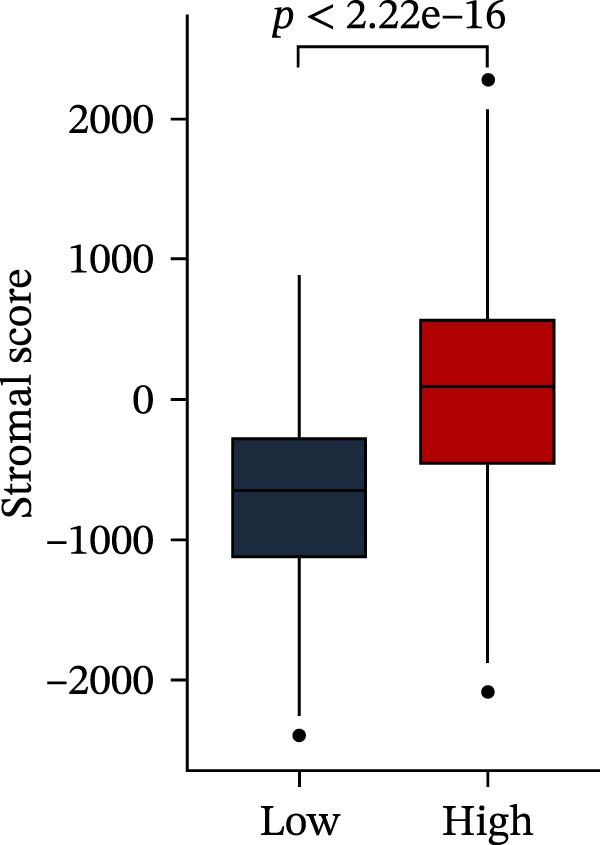
(D)

**Figure:**
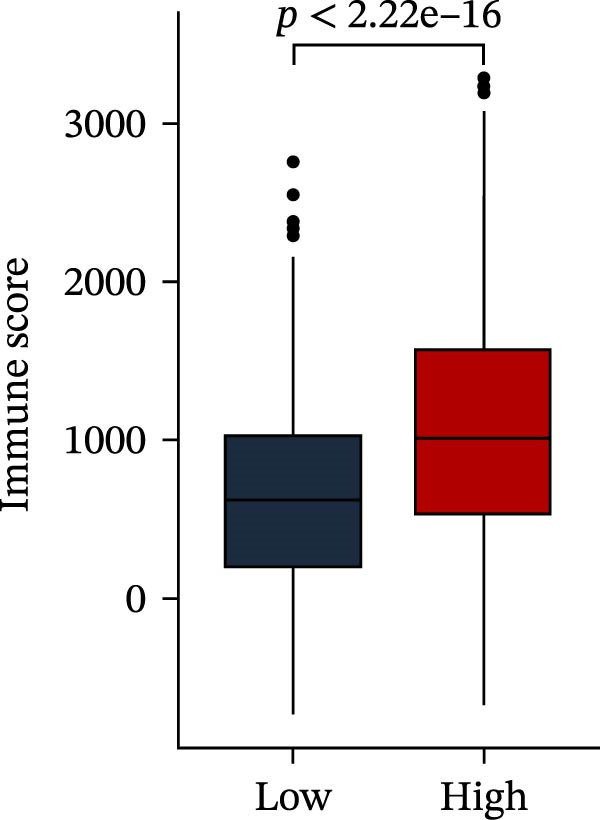
(E)

**Figure:**
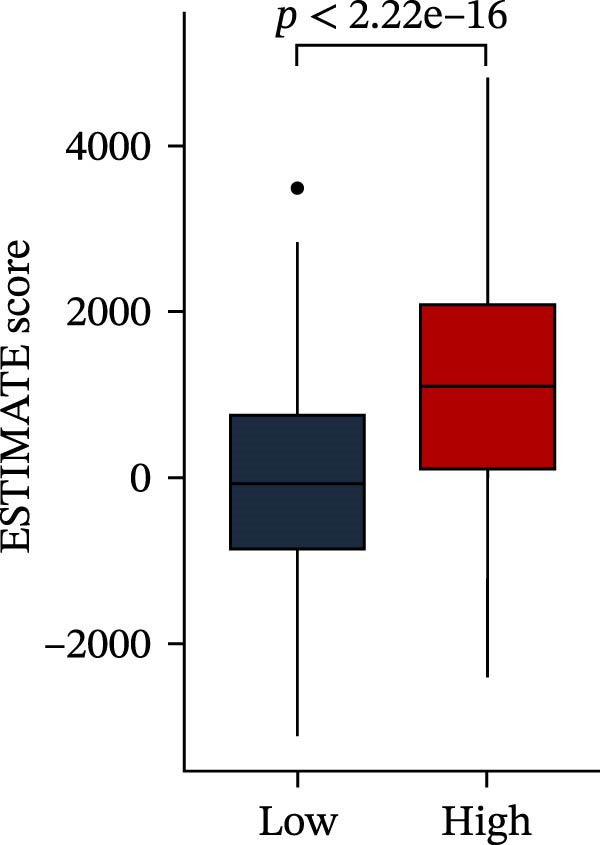
(F)

**Figure:**
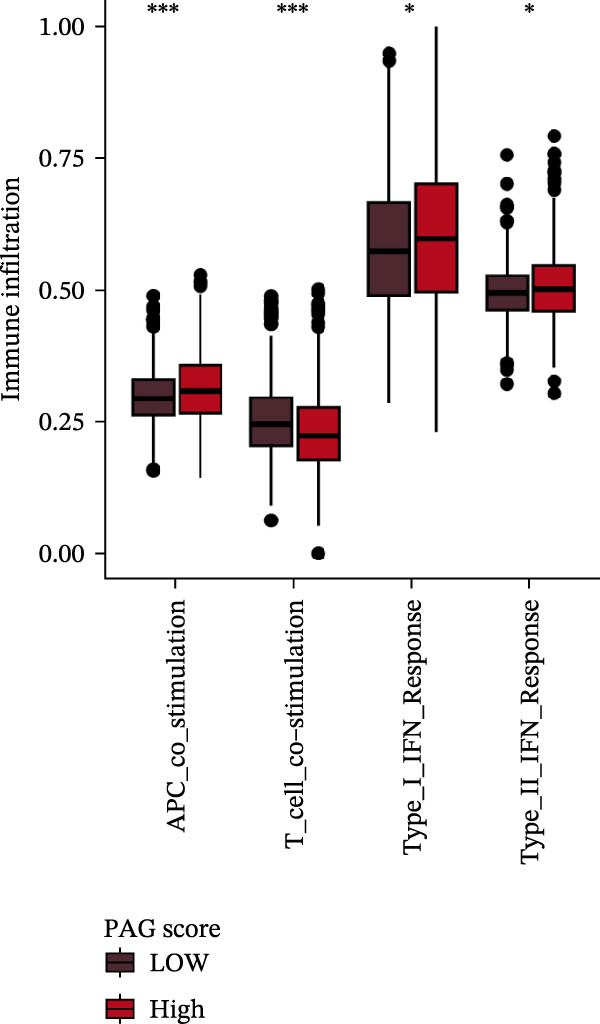
(G)

**Figure:**
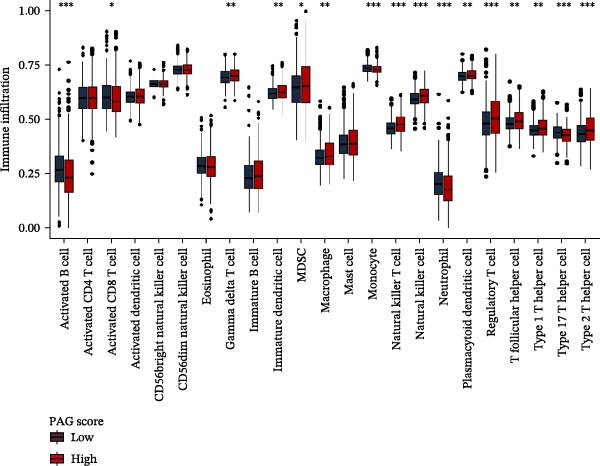
(H)

### 3.9. Mutational Landscape and Immunotherapy Response Prediction

MSI and tumor mutational burden are widely recognized as key determinants of immunotherapy response in COAD. In this study, the associations between the PAG score and MSI status as well as TMB were further evaluated. The results showed that PAG scores were significantly higher in the MSI‐H group than in the MSI‐L and MSS groups (Figure 10A, B). Consistently, the high PAG score subgroup exhibited significantly higher TMB levels (Figure 10C), suggesting a potential association between the PAG score and genomic instability in COAD. Immunotherapy response prediction suggested that the low PAG score subgroup may have a higher likelihood of benefiting from immunotherapy, as indicated by significantly lower TIDE scores (Figure 10D). Analysis of the IMvigor210 urothelial carcinoma cohort further demonstrated that patients with low PAG scores exhibited significantly better clinical outcomes following PD‐L1 blockade, suggesting a potential association that warrants further validation in COAD (Figure 10E). Moreover, in the IMvigor210 cohort, PAG scores were markedly lower in responders (CR/PR) than in nonresponders (SD/PD), further supporting a potential association between low PAG scores and improved immunotherapy response in urothelial carcinoma (Figure 10F). In addition, IPS analysis indicated significantly higher scores in the low PAG score subgroup, suggesting enhanced responsiveness to CTLA‐4 and PD‐1 immunotherapy (Figure 10G–J). Somatic mutation landscape analysis revealed that mutation frequencies of APC, KRAS, and PIK3CA were significantly lower in the high PAG score subgroup, whereas mutation frequencies of TTN, SYNE1, and MUC16 were significantly higher (Figure 10K, L), indicating distinct mutation patterns associated with PAG score stratification. Drug sensitivity analysis further demonstrated that the high PAG score subgroup exhibited significantly lower IC50 values for crizotinib, dasatinib, doxorubicin, imatinib, paclitaxel, pazopanib, saracatinib, and sunitinib, suggesting increased sensitivity to these agents (Figure 10M). Collectively, these findings suggest that the PAG score is closely associated with MSI status, TMB, driver gene mutation patterns, and therapeutic response, highlighting its potential exploratory value in guiding immunotherapy and chemotherapy strategies in COAD, though further clinical validation is needed.

Figure 10Somatic mutation landscape and immunotherapy response prediction analysis. (A, B) Differential analysis of PAG scores among MSS, MSI‐L, and MSI‐H subgroups. (C) TMB score analysis across PAG score subgroups. (D) TIDE score prediction of immunotherapy response. (E) Clinical survival curve analysis of PAG score subgroups constructed based on prognostic variables in the IMvigor210 cohort. (F) Differential analysis of PAG scores between CR/PR and SD/PD subgroups before and after PD‐L1 treatment. PR, partial response; PD, progressive disease; SD, stable disease; CR, complete response. (G–J) IPS score prediction revealing the response of PAG score subgroups to PD‐1 and CTLA‐4 immunotherapy. (K, L) Somatic mutation frequency analysis in PAG score subgroups. (M) Predicted IC50 analysis for drug sensitivity.

**Figure:**
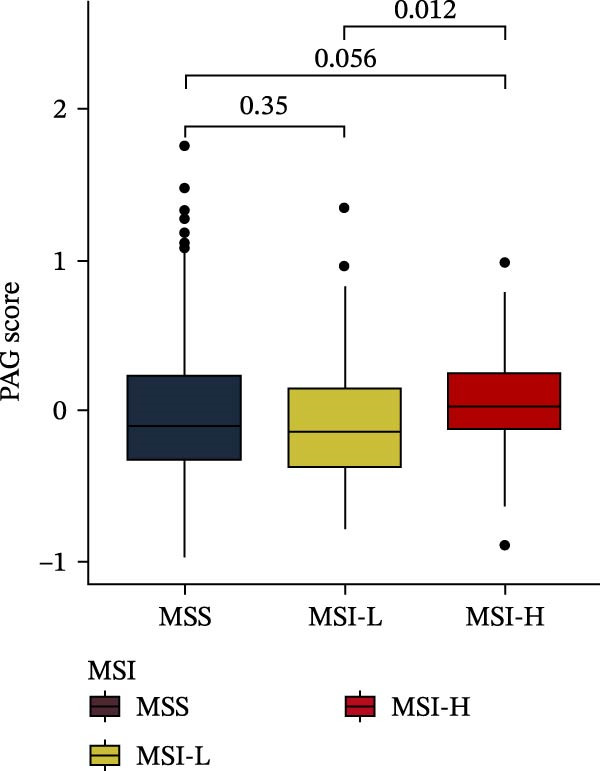
(A)

**Figure:**
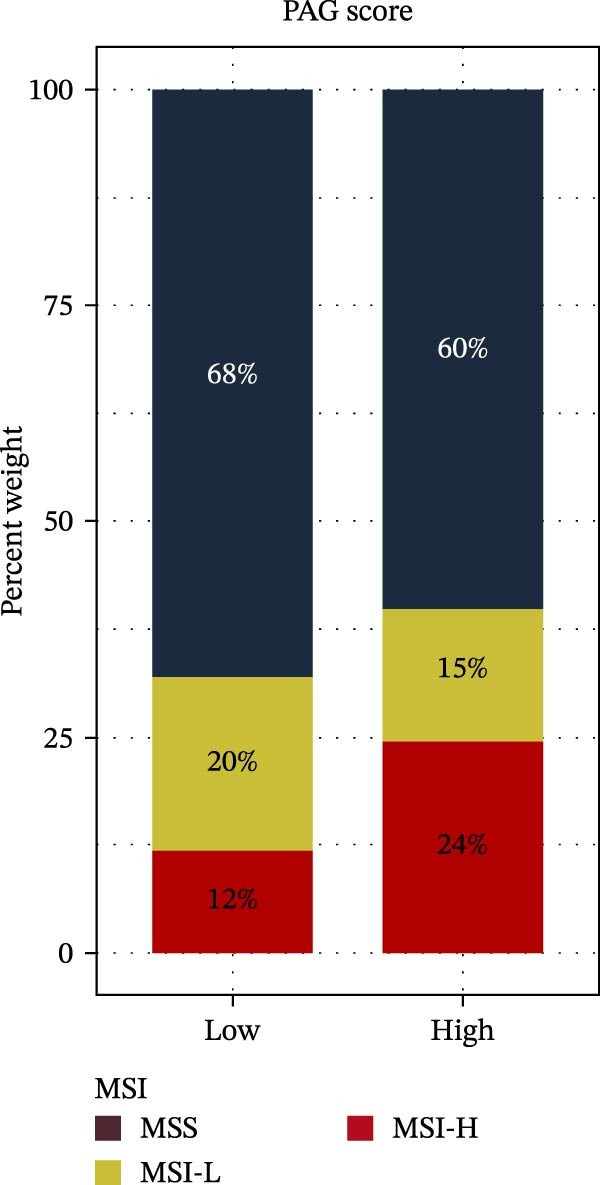
(B)

**Figure:**
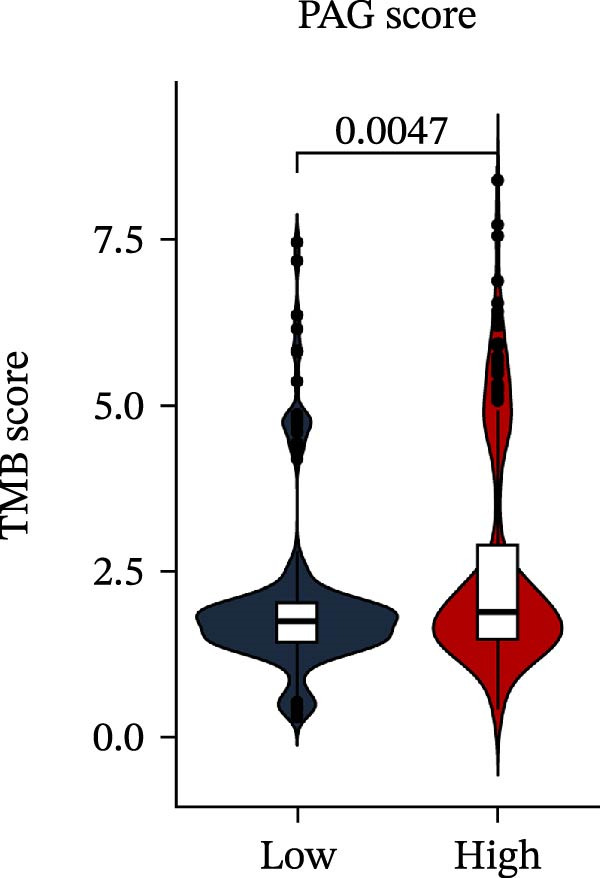
(C)

**Figure:**
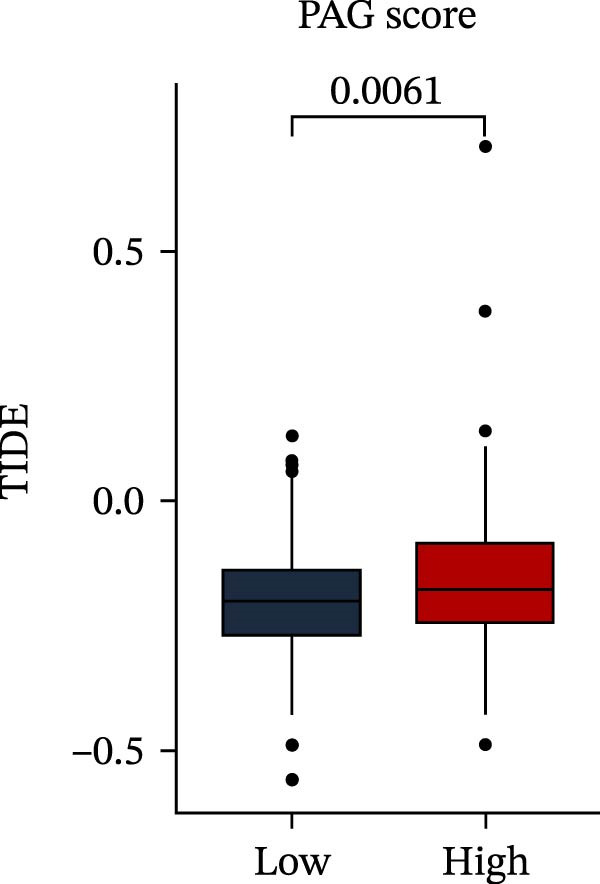
(D)

**Figure:**
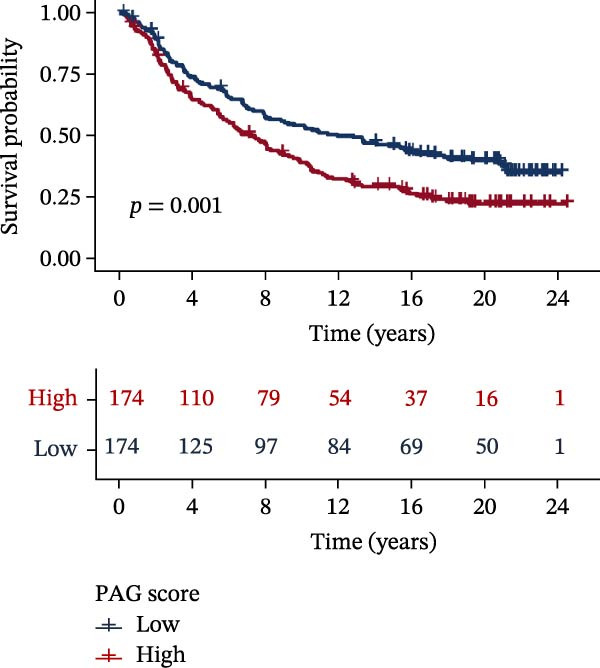
(E)

**Figure:**
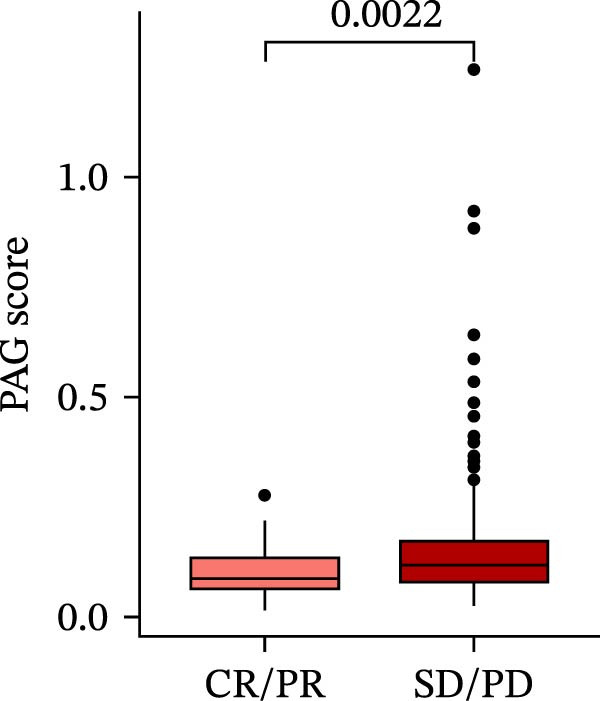
(F)

**Figure:**
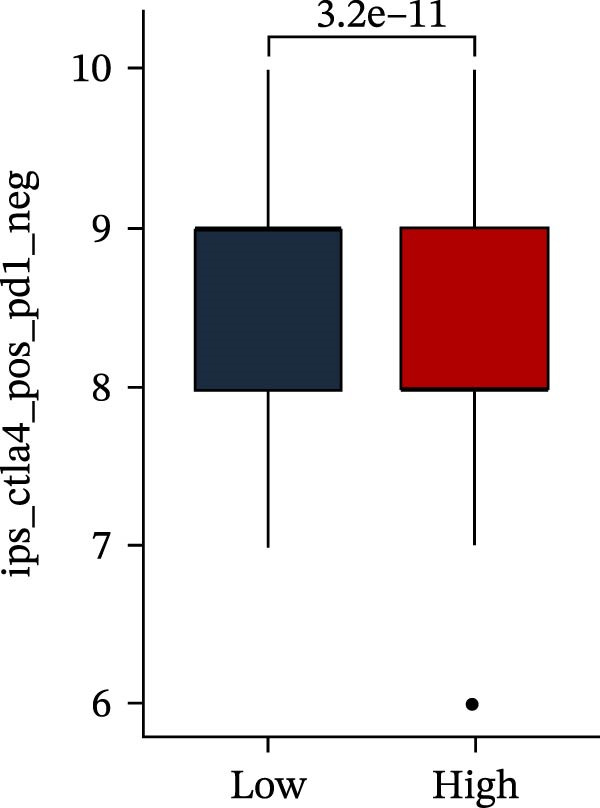
(G)

**Figure:**
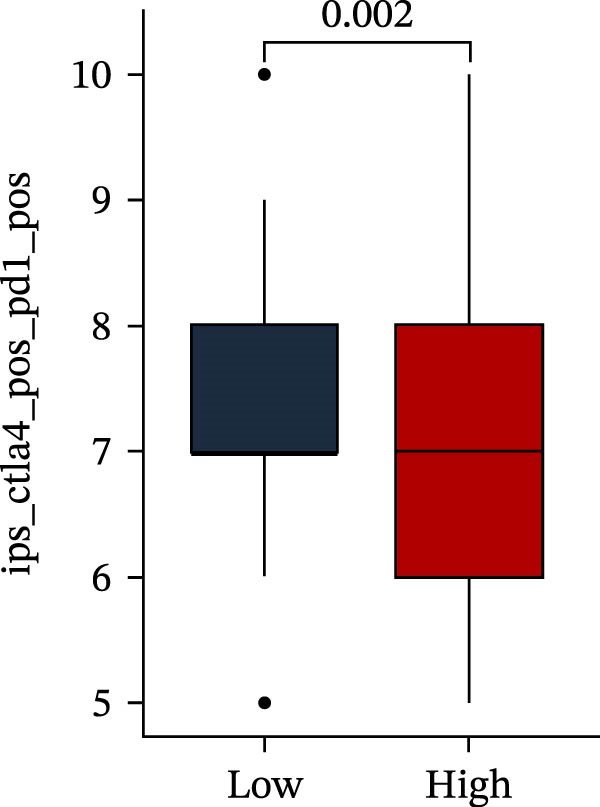
(H)

**Figure:**
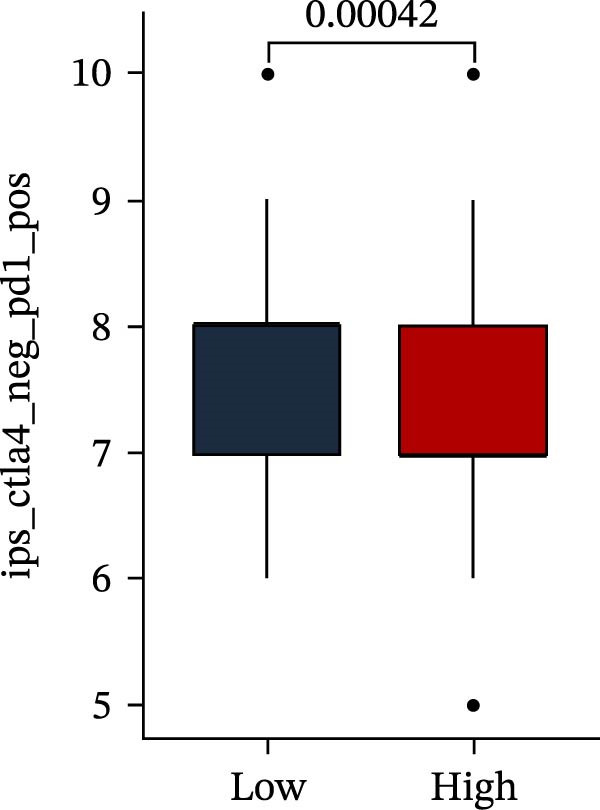
(I)

**Figure:**
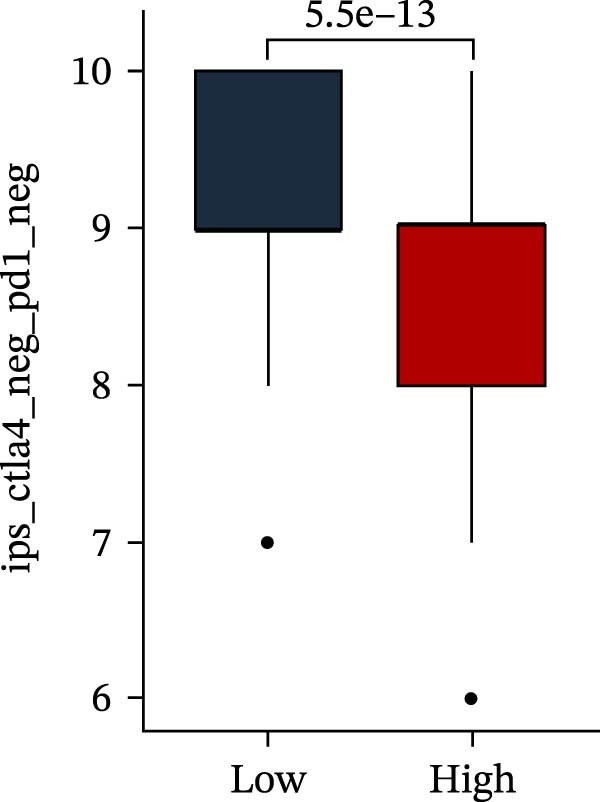
(J)

**Figure:**
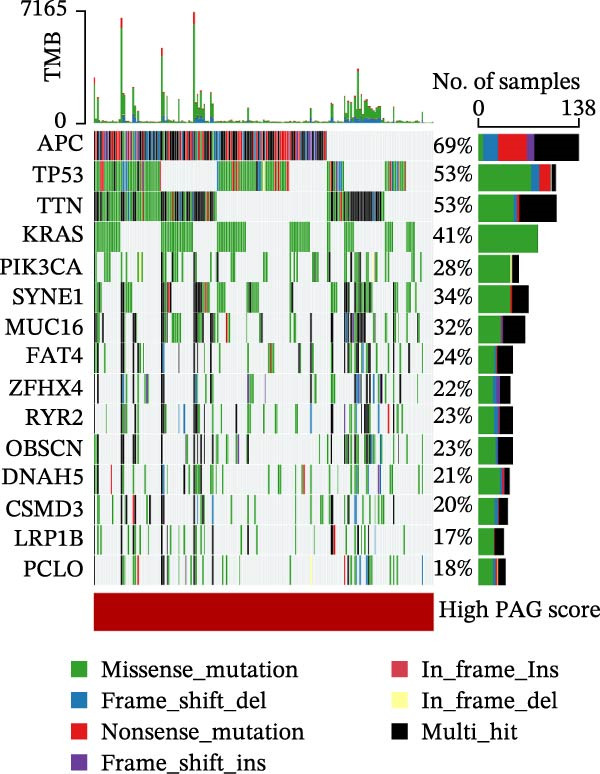
(K)

**Figure:**
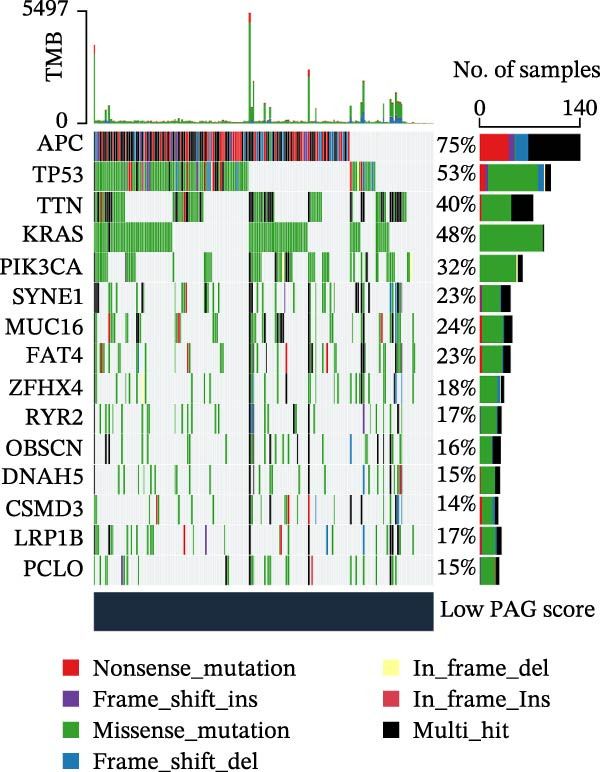
(L)

**Figure:**
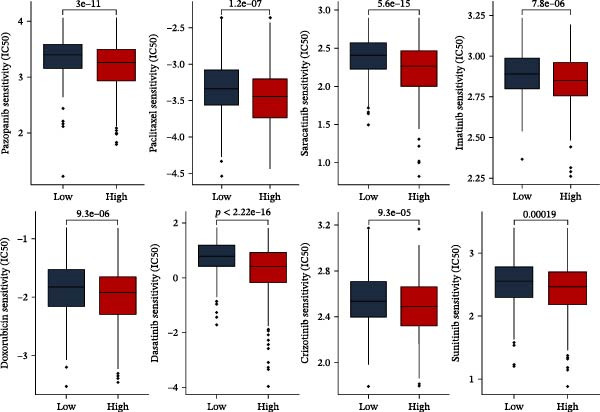
(M)

### 3.10. Single‐Cell RNA Sequencing Reveals Cellular Heterogeneity and Expression Patterns of Prognostic Signatures in COAD

To further explore cellular heterogeneity and the expression patterns of prognostic genes in COAD, single‐cell transcriptomic analysis was performed using the GSE231559 dataset, which included three normal tissue samples and three COAD tissue samples. After quality control, the top 2000 highly variable genes were selected for downstream dimensionality reduction analyses (Figure 11A, B). Batch effects were corrected using the Harmony algorithm to ensure data comparability across samples. Based on cell type–specific marker genes, 24 distinct cell clusters were accurately identified and visualized using UMAP and t‐SNE plots (Figure 11C, D). Violin plot analysis demonstrated that all 13 prognostic genes were significantly expressed across these cell clusters, with particularly prominent expression of SLC2A3 (Figure 11E). Using SingleR annotation, the 24 cell clusters were further consolidated into nine major cell types, including B cells, neutrophils, NK cells, dendritic cells, macrophages, endothelial cells, fibroblasts, epithelial cells, and ILCs. UMAP and t‐SNE projections illustrated the spatial distribution of these nine cell types (Figure 11F, G). PAG scoring revealed significant expression across all nine cell types (Figure 11H). Furthermore, UMAP‐based visualization showed the expression distributions of the 13 prognostic genes across the nine cell types, with SLC2A3 being relatively enriched in B cells and fibroblasts (Figure 11I–U). Overall, single‐cell RNA sequencing analysis revealed pronounced cellular heterogeneity in COAD and clarified the cell type–specific expression patterns of PAG‐related prognostic genes, providing valuable insights for future functional studies.

Figure 11Single‐cell RNA sequencing analysis identifying cell subpopulations and the expression distribution of prognostic signatures. (A) Quality control and normalization of single‐cell sequencing data (normal samples: 3 and tumor samples: 3). (B) Identification of the top 2000 highly variable genes. (C, D) UMAP and t‐SNE plots showing the distribution of 24 cell clusters. (E) Violin plots of prognostic signature expression across the 24 cell types. (F, G) UMAP and t‐SNE plots of nine annotated major cell subpopulations. (H) Enrichment scores of PAG in the nine cell subpopulations. (I–U) UMAP plots illustrating the expression distribution of prognostic signatures across the nine cell subpopulations.

**Figure:**
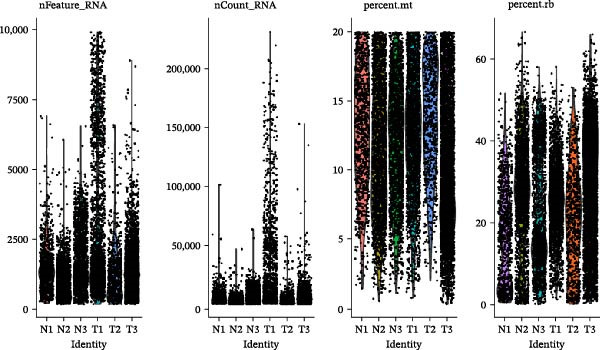
(A)

**Figure:**
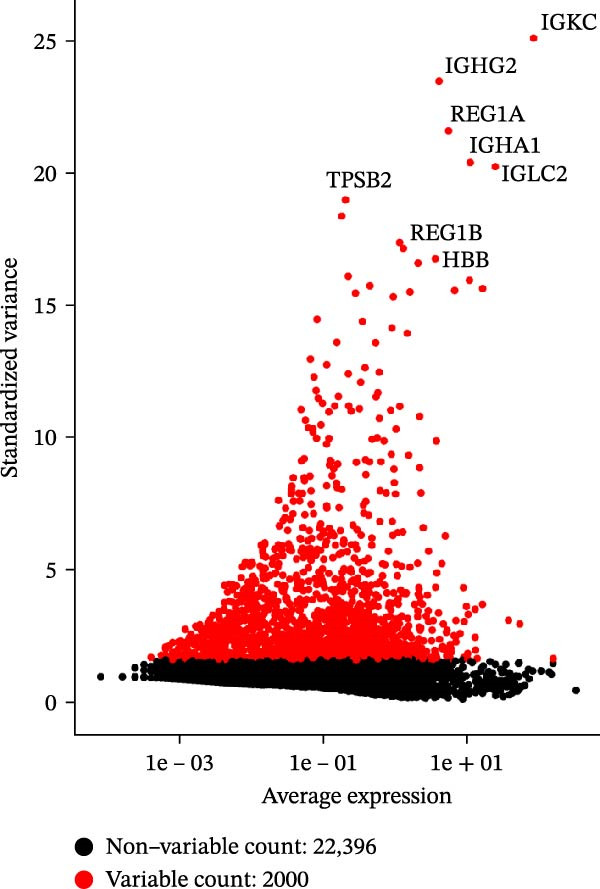
(B)

**Figure:**
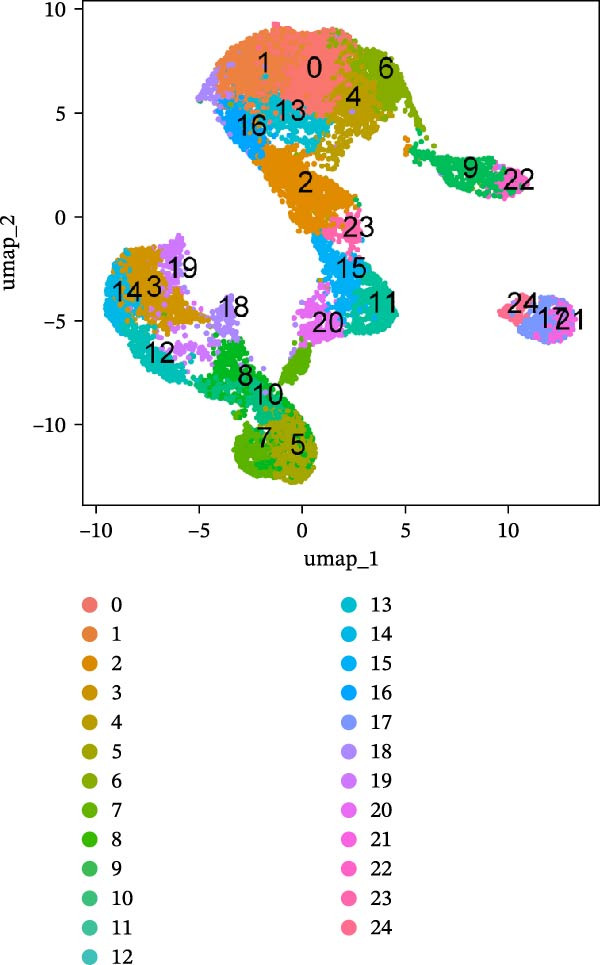
(C)

**Figure:**
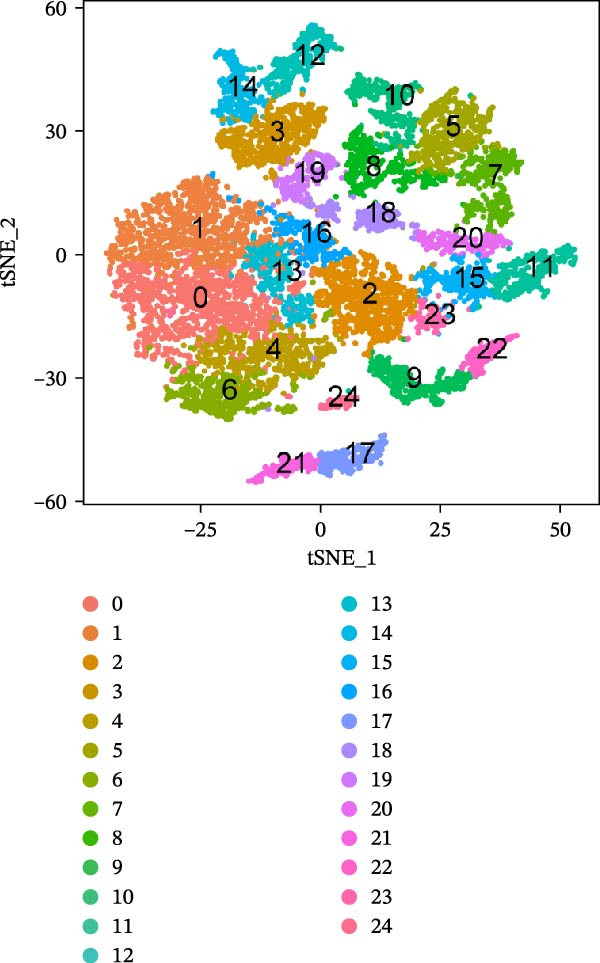
(D)

**Figure:**
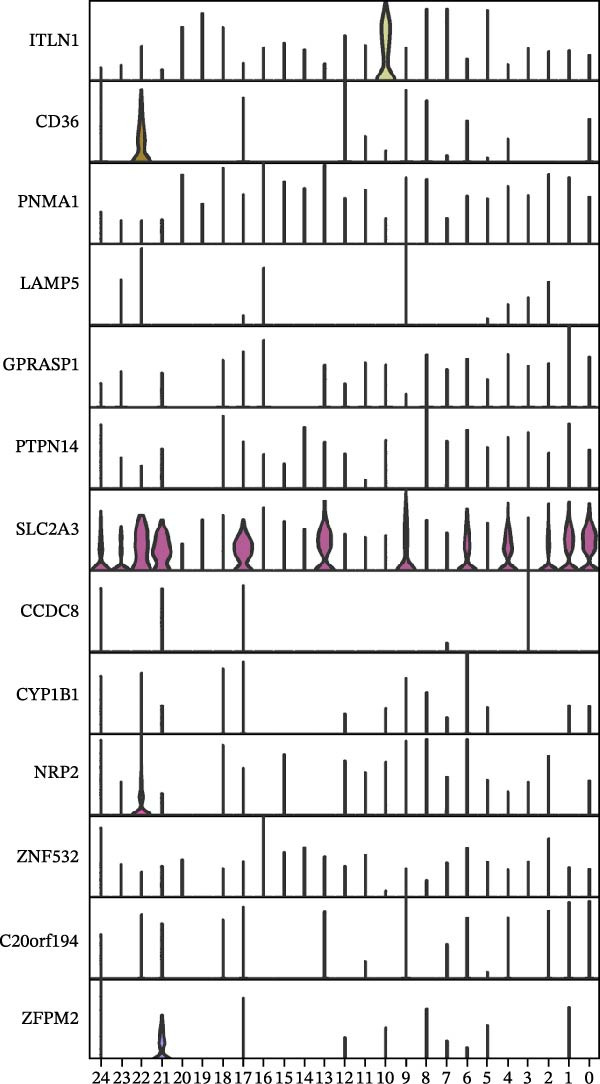
(E)

**Figure:**
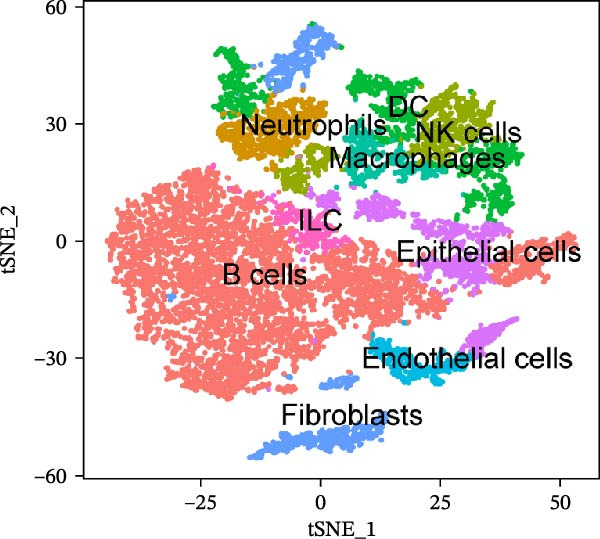
(F)

**Figure:**
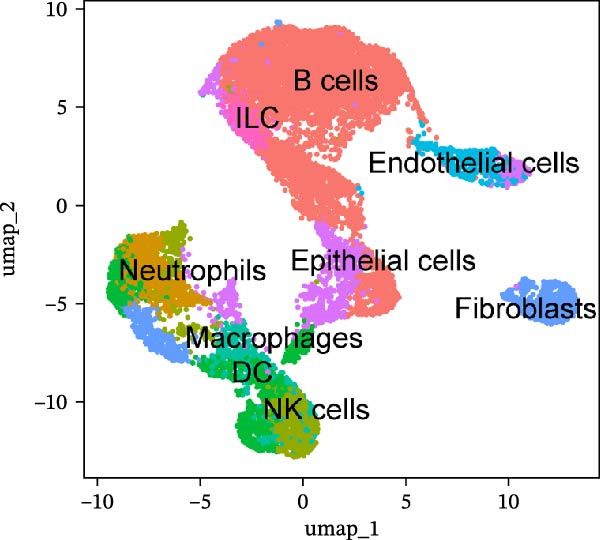
(G)

**Figure:**
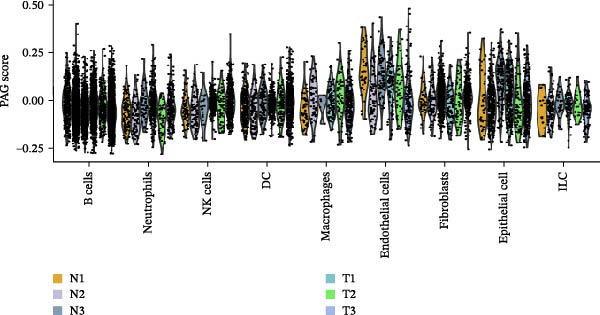
(H)

**Figure:**
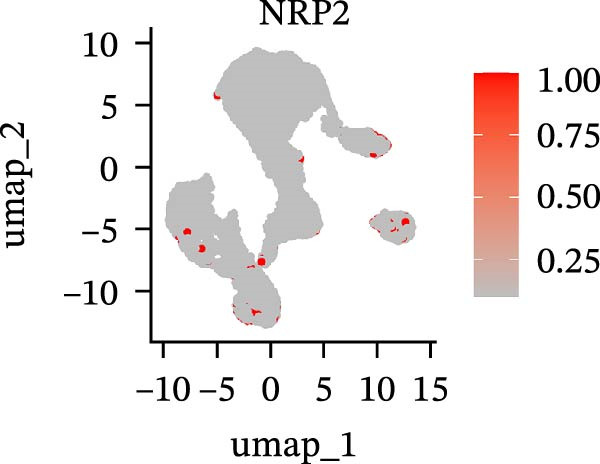
(I)

**Figure:**
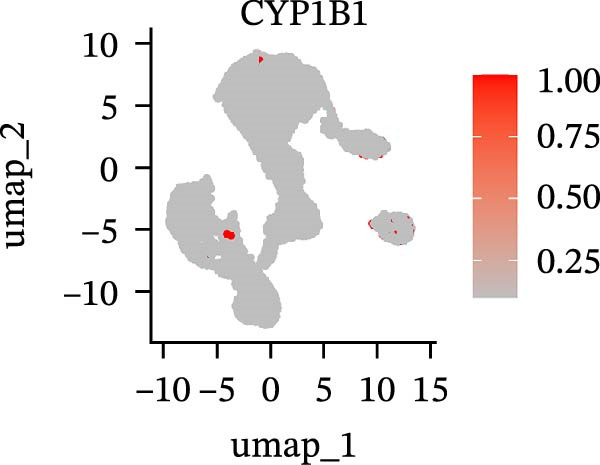
(J)

**Figure:**
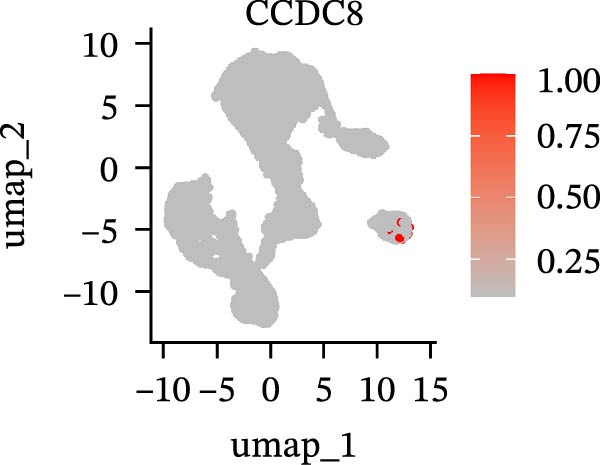
(K)

**Figure:**
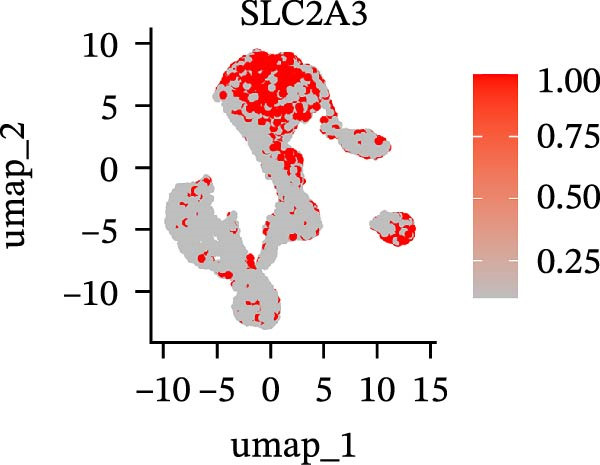
(L)

**Figure:**
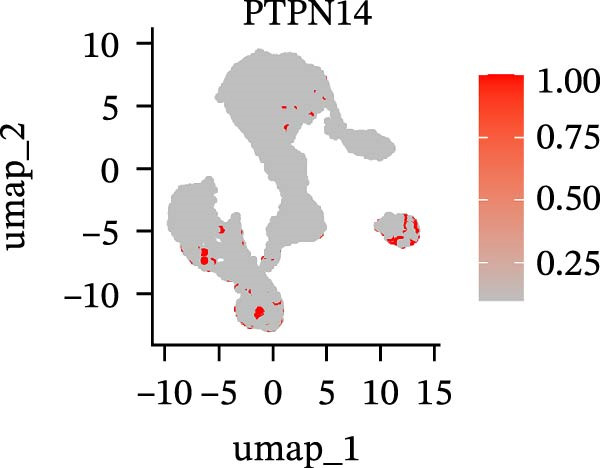
(M)

**Figure:**
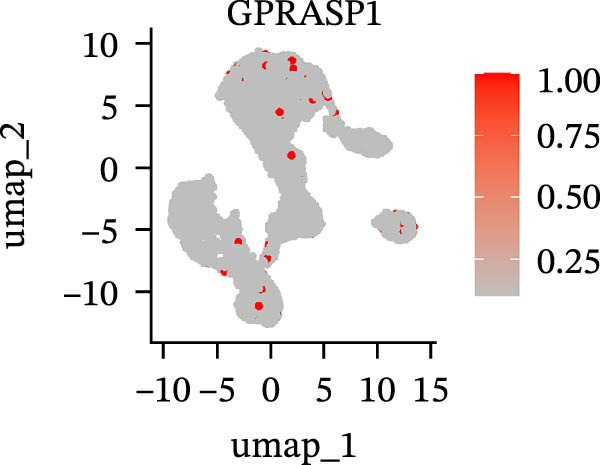
(N)

**Figure:**
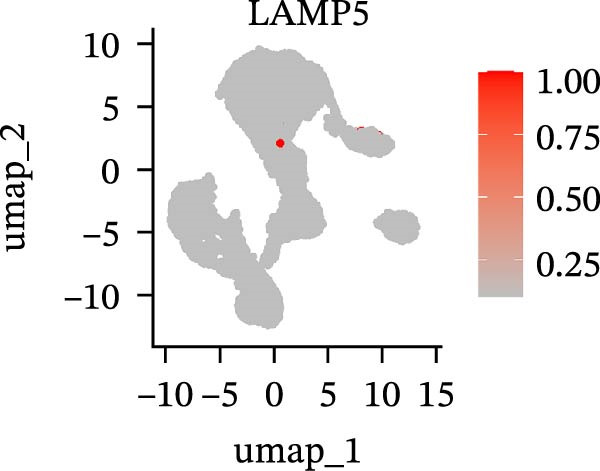
(O)

**Figure:**
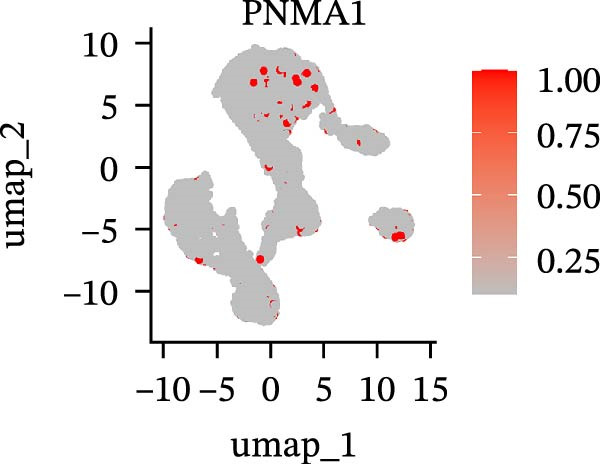
(P)

**Figure:**
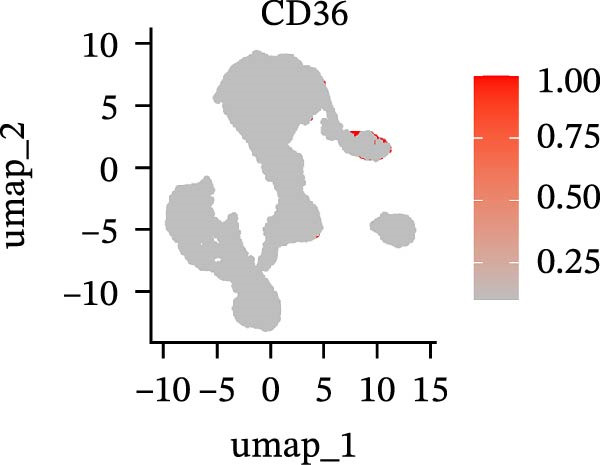
(Q)

**Figure:**
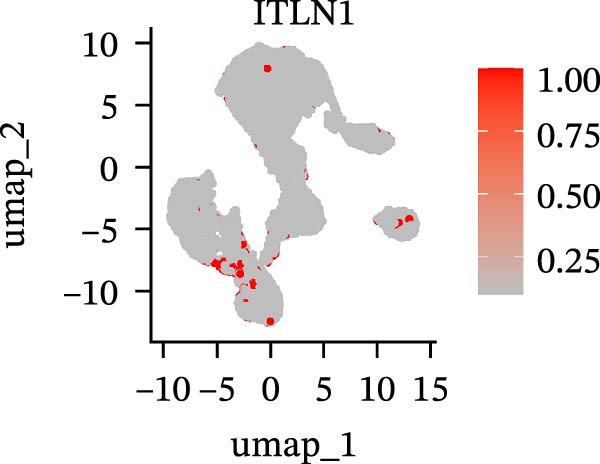
(R)

**Figure:**
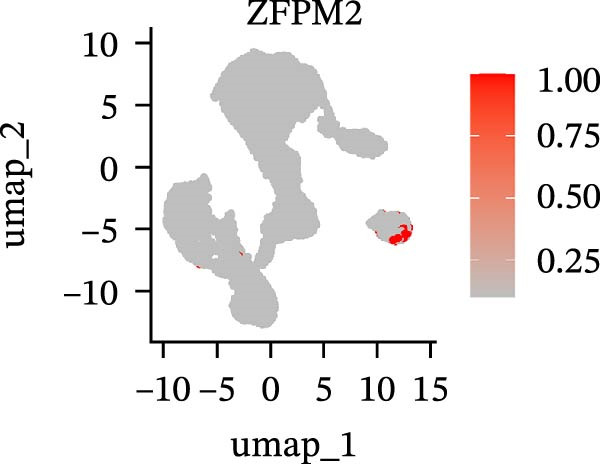
(S)

**Figure:**
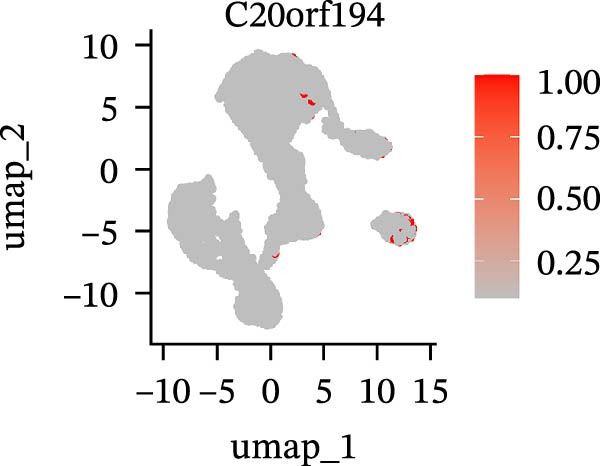
(T)

**Figure:**
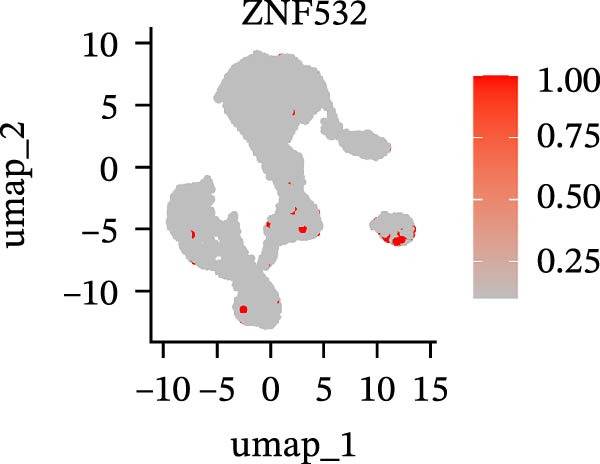
(U)

### 3.11. Functional Validation of SLC2A3 in COAD Cell Proliferation and Invasion

Within the PAG scoring system, SLC2A3 exhibited the highest risk coefficient, suggesting that it may serve as a key risk factor associated with poor prognosis in COAD. Survival analyses demonstrated that patients with low SLC2A3 expression had significantly better outcomes in terms of disease‐specific survival (DSS), OS, and progression‐free interval (PFI) compared with those with high expression levels, indicating a strong association between elevated SLC2A3 expression and unfavorable prognosis (Figure 12A–C). Differential and paired analyses further confirmed that SLC2A3 expression was significantly upregulated in COAD tissues compared with normal tissues (Figure 12D, E). Western blot analysis showed markedly higher SLC2A3 protein expression in the COAD cell line SW480 compared with the normal colon epithelial cell line NCM460 (Figure 12F). To further investigate the functional role of SLC2A3 in COAD cells, an siRNA‐mediated knockdown model was established in SW480 cells. Knockdown efficiency analysis confirmed a significant reduction in SLC2A3 protein expression following siRNA transfection (Figure 12G). Functional assays suggested that SLC2A3 knockdown was associated with reduced cell proliferation and invasion capabilities in SW480 cells (Figure 12H, I). CCK‐8 assays further revealed significantly reduced cell viability at 24, 48, 72, and 96 h in the siSLC2A3 group compared with the siNC group (Figure 12J). In summary, these findings identify SLC2A3 as a key risk gene associated with poor prognosis in COAD. Preliminary in vitro experiments using the SW480 cell line suggest that SLC2A3 knockdown may suppress tumor cell proliferation and invasion, supporting its potential as a candidate for further investigation. Nevertheless, comprehensive functional validation across multiple cell lines and in vivo models is required to establish its mechanistic role and therapeutic relevance.

Figure 12Knockdown of SLC2A3 significantly inhibits COAD cell proliferation and invasion. (A–C) Clinical survival curves stratified by high and low SLC2A3 expression. DSS, disease‐specific survival; OS, overall survival; PFI, progression‐free interval. (D) Differential expression analysis of SLC2A3 between normal and tumor groups. (E) Paired analysis of SLC2A3 expression levels between normal and tumor groups. (F) Protein expression and quantification analysis of SLC2A3 in NCM460 and SW480 cell lines (*n* = 3). (G) Protein expression and quantification analysis of SLC2A3 before and after knockdown (*n* = 3). (H) Colony formation assay analysis (*n* = 3). (I) Transwell invasion assay analysis (*n* = 3). (J) CCK‐8 cell viability assay at 0, 24, 48, 72, and 96 h (*n* = 3). Data are presented as mean ± SD;  ^∗^ *p* < 0.05,  ^∗∗^ *p* < 0.01, and  ^∗∗∗^ *p* < 0.001.

**Figure:**
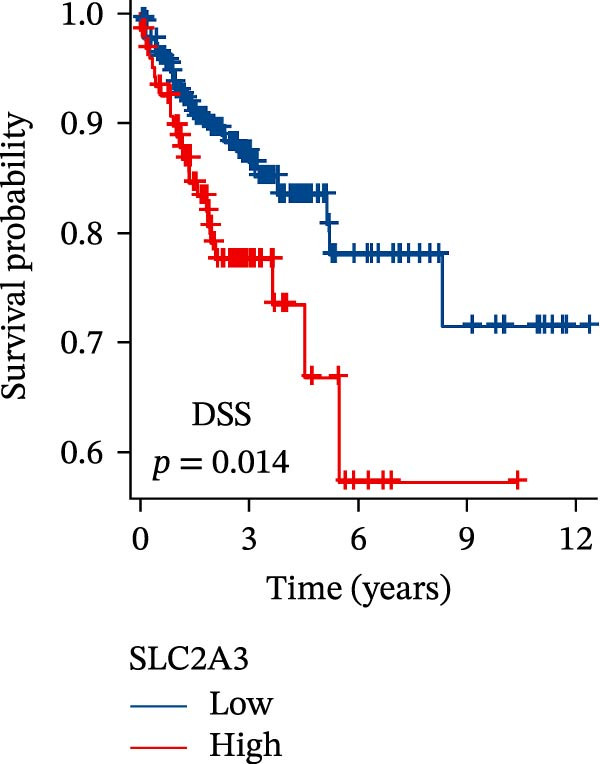
(A)

**Figure:**
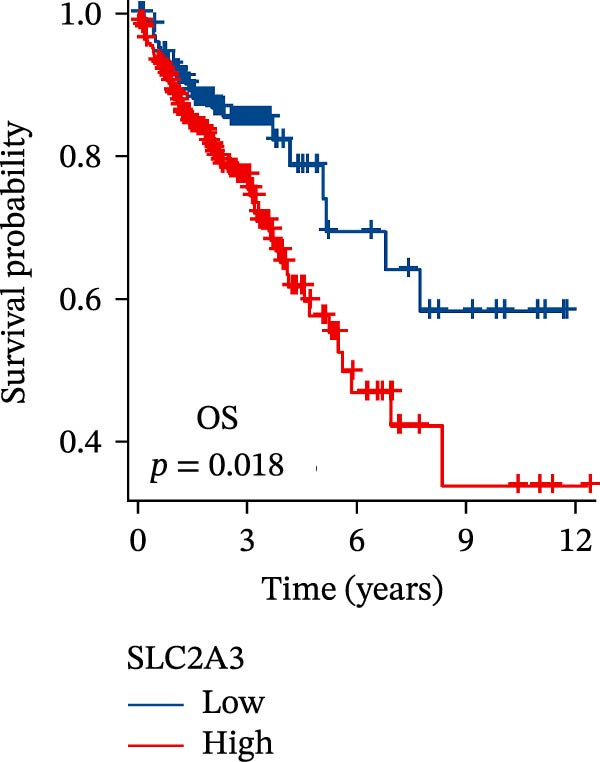
(B)

**Figure:**
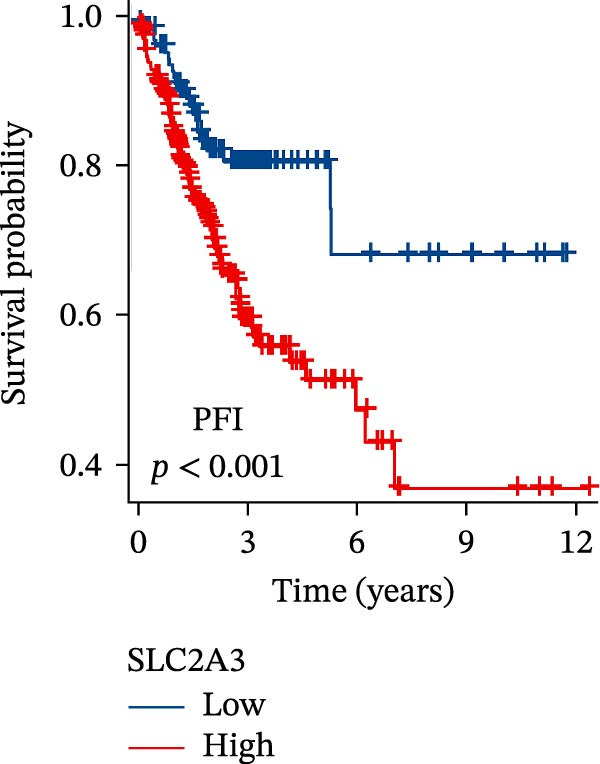
(C)

**Figure:**
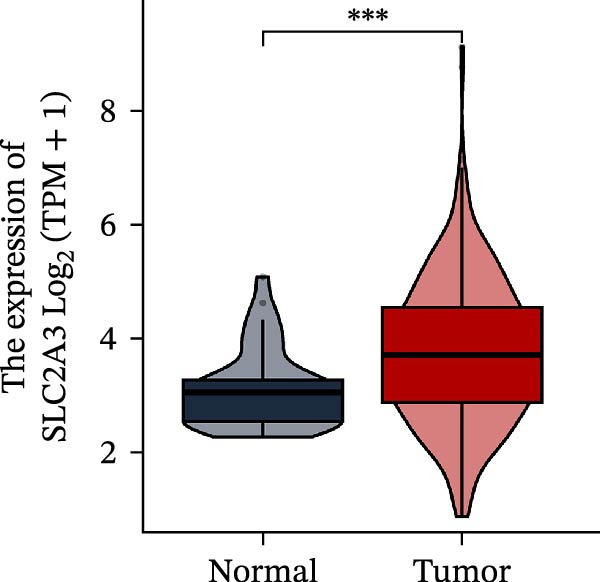
(D)

**Figure:**
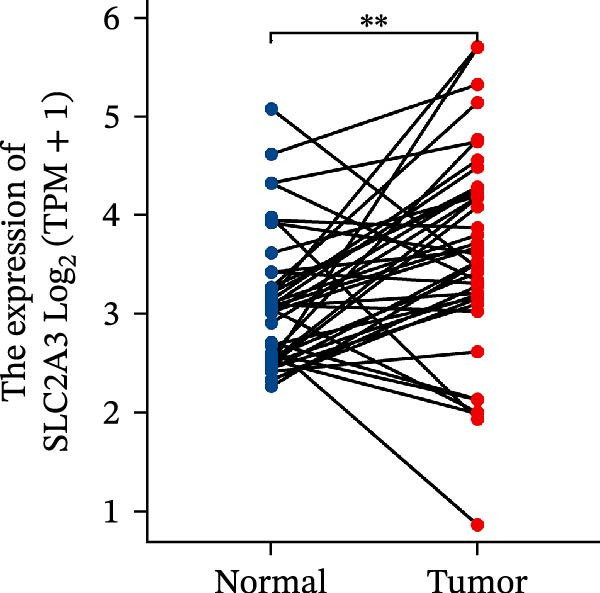
(E)

**Figure:**
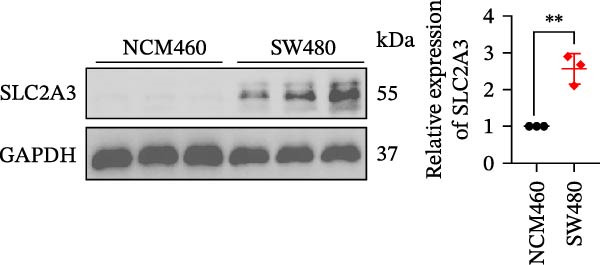
(F)

**Figure:**
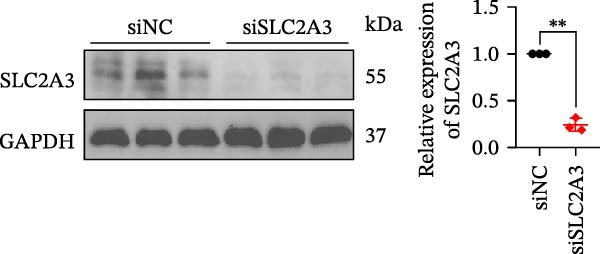
(G)

**Figure:**
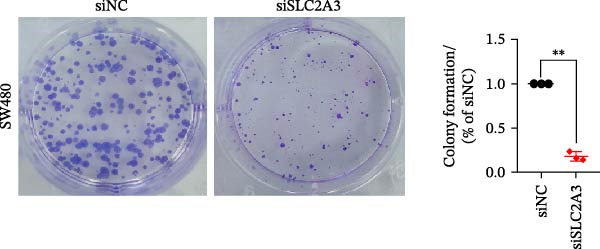
(H)

**Figure:**
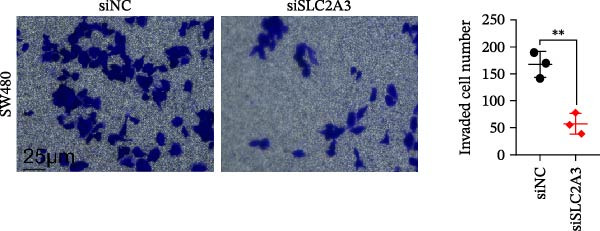
(I)

**Figure:**
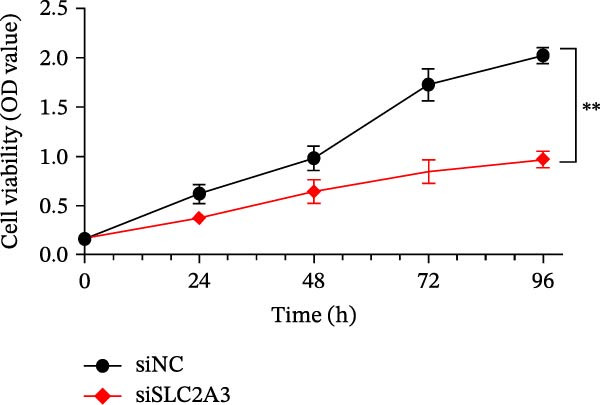
(J)

## 4. Discussion

Our results suggest a potential role of parthanatos in the initiation and progression of COAD. Although comprehensive evidence remains limited, several independent studies have provided clues implicating parthanatos in CRC, including COAD. For instance, inhibition of autophagy in CRC cells has been shown to induce cell death through the activation of parthanatos [17]. Similarly, suppression of the oncogenic AKT signaling pathway can trigger parthanatos‐mediated cell death in CRC cells, highlighting parthanatos as a potential therapeutic strategy for COAD [18]. Moreover, combining parthanatos‐targeted interventions with standard chemotherapy significantly prolongs survival in colon cancer xenograft mouse models [19, 20]. Parthanatos has also been implicated in the regulation of drug resistance in CRC cells [21, 22]. Accordingly, therapeutic strategies that incorporate modulation of parthanatos may help overcome multidrug resistance and suppress tumor progression [20].

Our in vitro experiments further confirmed that high expression of SLC2A3 in COAD is associated with poor prognosis and that knockdown of SLC2A3 significantly inhibits COAD cell proliferation and invasion. As a glucose transporter, SLC2A3 exhibits high affinity for glucose and facilitates its transport from the extracellular milieu into the cytoplasm [23]. During aerobic glycolysis, tumor maintenance and malignant progression are often supported by the increased energy supply driven by upregulated SLC2A3 expression [24–26]. In addition, a wide range of tumor biological behaviors, including proliferation, migration, invasion, distant metastasis, and chemoresistance, have been reported to be associated with SLC2A3 [27]. In CRC, SLC2A3 is also closely involved in aerobic glycolysis and metabolic reprogramming [28, 29]. Consistently, SLC2A3 has been linked to multiple biological features of COAD, including tumorigenesis, metastasis, drug resistance, epithelial‐mesenchymal transition (EMT), and immune‐related characteristics [30–33]. Integrating these findings with our multiomics data suggests a cohesive model linking SLC2A3 to parthanatos and the tumor microenvironment. Parthanatos is triggered by PARP1 hyperactivation following severe DNA damage, leading to NAD^+^/ATP depletion and an energy crisis [14, 34]. To survive, cancer cells may upregulate SLC2A3 to enhance glucose flux, replenish NAD^+^, and sustain glycolysis, thereby resisting parthanatos [35]. This metabolic adaptation also shapes immune modulation: Altered glucose metabolism causes lactate accumulation and nutrient competition, suppressing effector immune cells while promoting immunosuppressive populations [30, 35]. PARP1 activation itself can influence immune‐related gene expression [14]. Thus, SLC2A3‐mediated metabolic reprogramming may bridge parthanatos resistance and the immunosuppressive microenvironment observed in high PAG score patients, consistent with reports linking SLC2A3 to EMT and immune signatures in CRC. Collectively, SLC2A3 emerges as a potential hub connecting parthanatos, metabolism, and immune evasion in COAD, warranting further investigation as a therapeutic target [36].

Beyond SLC2A3‐associated differences in drug resistance, other PAG‐related processes may also contribute to heterogeneous therapeutic responses [13], supporting the clinical relevance of targeting parthanatos. In current clinical practice, resistance driven by p53 mutations and overexpression of the antiapoptotic protein Bcl2 largely arises from reliance on caspase‐dependent conventional therapies [37]. Accordingly, many therapeutic strategies have focused on p53‐ and Bcl2‐related antiapoptotic pathways [38]. Notably, the ability of cancer cells to evade apoptosis while remaining susceptible to parthanatos has been demonstrated in multiple experimental models [39]. Our results showed that doxorubicin, a representative proapoptotic drug acting through the classical p53‐BAX‐caspase pathway, exhibited IC50 values that were significantly associated with PAG stratification, suggesting a potential synergistic interaction between parthanatos and conventional proapoptotic therapies [40]. Importantly, in tumors that are refractory to apoptosis‐based treatments, targeting parthanatos may still exert effective antitumor activity, making it a promising alternative therapeutic approach [41].

Our immune infiltration analysis revealed that plasmacytoid dendritic cells (pDCs) were significantly enriched in the PAG high‐score group and were associated with poorer prognosis. Unlike other immune cell populations that showed marked alterations, such as regulatory T cells (Tregs), NK T cells, or NK cells, the role of pDCs in tumors appears to be highly complex and context dependent [42]. Owing to their unique capacity to produce type I IFNs and modulate immune responses within the tumor microenvironment, pDCs play a pivotal role in the intricate interplay between the immune system and cancer [43]. Some studies have reported that pDCs exert antitumor effects by promoting the activation of cytotoxic T cells and NK cells with tumor‐suppressive functions [44, 45]. Conversely, other studies have demonstrated that pDCs can induce Tregs and enhance the production of immunosuppressive cytokines, thereby facilitating immune tolerance, tumor immune evasion, and disease progression [46]. In CRC, including COAD, pDCs have been reported to promote Treg development within immunotolerant tumor microenvironments [47]. However, contrasting evidence suggests that tumor‐infiltrating pDCs may correlate with improved survival in human colon cancer by producing higher levels of antitumor IFN‐α [48]. Collectively, these findings highlight the necessity of further elucidating the role of pDCs across different tumor types and at distinct stages of tumor progression.

Several methodological limitations should be acknowledged. First, although we employed an integrative machine learning framework with 64 algorithm combinations under LOOCV to minimize overfitting, the potential for overfitting cannot be completely excluded given the high‐dimensional nature of transcriptomic data and the relatively modest sample size [49]. Feature selection stability varied across algorithms, underscoring the value of ensemble strategies [50], yet the model offers limited inherent interpretability regarding biological relationships [51]. Second, the PAGs were obtained from the GeneCards database based on relevance scores and reported associations, which may introduce genes with indirect or less well‐established links to parthanatos and thus affect the specificity of the gene set. Third, our conclusions regarding immunotherapy responsiveness and immune microenvironment characteristics rely on computational tools, including ESTIMATE, ssGSEA, TIDE, and IPS, which are based on transcriptomic inference and may be influenced by data quality, tumor heterogeneity, and algorithmic assumptions [52, 53]. Although differences between groups were evaluated using the Wilcoxon rank‐sum test, these findings remain predictive rather than definitive, and the use of the IMvigor210 cohort provides only hypothesis‐generating evidence requiring further validation in COAD‐specific immunotherapy cohorts [54, 55]. Finally, functional validation of SLC2A3 was performed in a single CRC cell line, which limits generalizability [56]; future studies should include multiple models and in vivo validation to confirm its biological role.

In summary, our study delineates a previously underappreciated role of parthanatos in the development, progression, and therapeutic response of COAD. By integrating transcriptomic profiling, prognostic modeling, immune infiltration analysis, and experimental validation, we demonstrate that parthanatos‐related programs are closely linked to tumor metabolism, drug sensitivity, and immune regulation. In particular, SLC2A3 emerges as a key PAG associated with poor prognosis in COAD, with preliminary in vitro evidence suggesting a potential role in promoting tumor cell proliferation and invasion that warrants further investigation.

## Author Contributions

Lei Luo created the study concept and design. Zhenyuan Zhao and Kun Ju conceived the original ideas and composed this manuscript. Jiajun Lv contributed to the tables and figures in this manuscript. Zidong Lu completed the experimental design and data analysis.

## Funding

The authors have nothing to report.

## Disclosure

All authors contributed to the article and approved the submitted version.

## Ethics Statement

The authors have nothing to report.

## Consent

The authors have nothing to report.

## Conflicts of Interest

The authors declare no conflicts of interest.

## Supporting Information

Additional supporting information can be found online in the Supporting Information section.

## Supporting information


**Supporting Information 1** Table S1: The gene list of parthanatos‐associated genes.

**Supporting Information 2** Figure S1: Identification of PAG‐related molecular subtypes.

## Data Availability

The data that support the findings of this study are available from the corresponding author upon reasonable request.
