# Supplementary material for: Identification and Experimental Verification Reveal SLC2A3 Associated With Prognosis and Immune Infiltration in Colon Adenocarcinoma
Source: Mediators Inflamm. 2026 Apr 24;2026:8383379. doi: 10.1155/mi/8383379 (PMC13107956; doi:10.1155/mi/8383379)
Supplement: Supplementary file 2 — Supporting Information 2 Figure S1: Identification of PAG‐related molecular subtypes. [file MI-2026-8383379-s001.pdf]

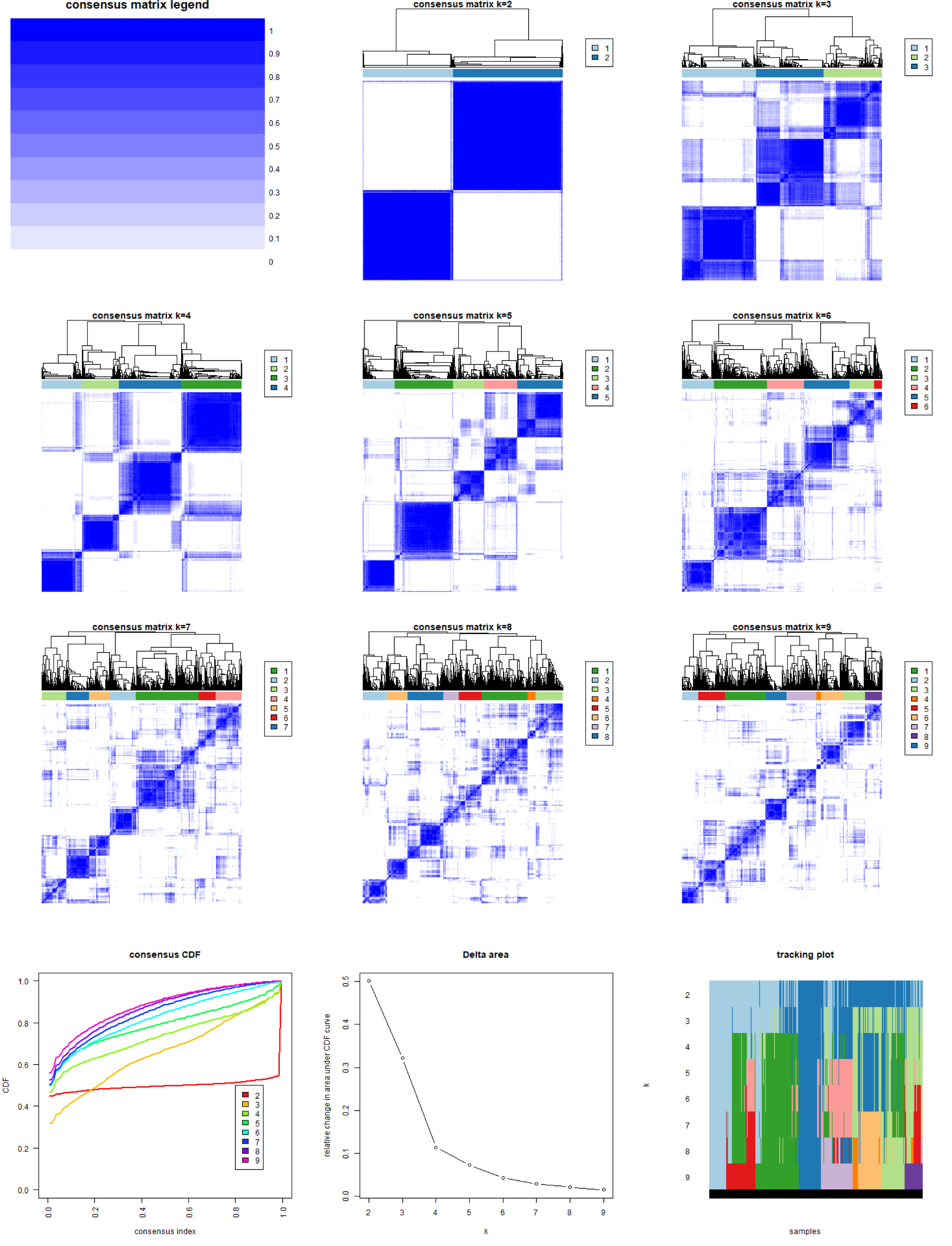
consensus matrix legend
1
0.9
0.8
0.7
0.6
0.5
0.4
0.3
0.2
0.1
0
consensus matrix k=2
consensus matrix k=3
consensus matrix k=4
consensus matrix k=5
consensus matrix k=6
consensus matrix k=7
consensus matrix k=8
consensus matrix k=9
consensus CDF
CDF
consensus index
Delta area
relative change in area under CDF curve
k
tracking plot
samples
